# Bioengineering a Patient‐Derived Vascularized Lung Tumor‐on‐Chip Model to Decipher Immunomodulation by the Endothelium

**DOI:** 10.1002/adhm.202403446

**Published:** 2025-05-09

**Authors:** Christine Lansche, Ségolène Ladaigue, Giacomo Gropplero, Nicolas Zimmermann, Martin Nurmik, Irina Veith, Manh‐Louis Nguyen, Solenn Brosseau, Nicolas Poté, Pierre Mordant, Arnaud Roussel, Fathia Mami‐Chouaib, Fatima Mechta‐Grigoriou, Gérard Zalcman, Fabrice Soncin, Stéphanie Descroix, Maria Carla Parrini

**Affiliations:** ^1^ Institut Curie U1339 INSERM – UMR3666 CNRS Stress and Cancer Laboratory PSL Research University Paris 75005 France; ^2^ Institut Curie CNRS UMR168 Laboratoire Physico Chimie Curie Institut Pierre‐Gilles de Gennes PSL Research University Paris 75005 France; ^3^ CIC INSERM 1425 Thoracic Oncology Department Hospital Bichat‐Claude Bernard, Université Paris Cité Paris 75018 France; ^4^ INSERM UMR1152 Department of Pathology Hospital Bichat‐Claude Bernard, Université Paris Cité Paris 75018 France; ^5^ INSERM UMR1152 Department of Vascular Surgery Thoracic Surgery and Lung Transplantation Hospital Bichat‐Claude Bernard, Université Paris Cité Paris 75018 France; ^6^ INSERM UMR 1186 Integrative Tumor Immunology and Immunotherapy Gustave Roussy Fac. de Médecine – Univ. Paris‐Sud Université Paris‐Saclay Villejuif 94800 France; ^7^ CNRS/IIS/Centre Oscar Lambret/Lille University SMMiL‐E Project CNRS Délégation Hauts‐de‐France Lille 59000 France; ^8^ CNRS IRL2820 Laboratory for Integrated Micro Mechatronic Systems Institute of Industrial Science University of Tokyo Tokyo 113‐8654 Japan

**Keywords:** cancer models, endothelium, immunomodulation, microfabrication, tumor microenvironment, tumor‐on‐chip

## Abstract

The endothelium compartment is a key player in tumor initiation and progression, but most existing tumor‐on‐chip models lack clinical relevance. Here, a 3D vascularized tumor‐on‐chip (vToC) model, generated with patient‐derived microvascular endothelial cells (ECs) that are freshly isolated from surgical lung cancer samples, is presented. The microvessel molecular identity, morphology, and functionality are assessed by transcriptomic, immunofluorescence, TNF‐α stimulation, and permeability assays. Lung cancer cells, cancer‐associated fibroblasts (CAFs), and CD8+ tumor‐infiltrating lymphocytes are embedded into the surrounding collagen matrix to partially recapitulate the lung tumor microenvironment (TME). The proof‐of‐concept of feasibility to generate personalized immunocompetent vToC composed of primary fully autologous cell types is provided. This vToC model is used to investigate the interplay between ECs and other TME cellular components by transcriptomic analysis. Using a rationally designed panel of endothelial genes, it is found that the presence of cancer cells and CAFs in the endothelial environment decreases expression by ECs of VCAM‐1 leukocyte adhesion protein, a crucial regulator of immune infiltration, and of many immunomodulatory chemokines, recapitulating endothelial cell anergy. This in vitro model will be a valuable clinically‐relevant tool to study the tumor‐CAF‐immune–endothelium interplay.

## Graphical abstract

Most in vitro cancer models lack cellular diversity, functional complexity, and clinical relevance. This work highlights the generation of an innovative dynamic tetraculture with autologous patient‐derived cells within a vascularized tumor‐on‐chip, as well as the recapitulation of endothelial anergy features induced by the tumor microenvironment.

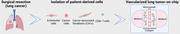

## 1 Introduction

In recent years, the organ‐on‐a‐chip (OoC) technology has emerged as a promising alternative to animal experimentation for in vitro recapitulation of key biological processes and for preclinical drug‐testing, with greater predicting power when compared to conventional 2D cell culture models.^[^ ^1^ ^]^ OoC systems, also named microphysiological systems, consist of micro‐engineered devices containing 3D biomimetic matrixes and living cell populations with appropriate features (cell identity, compartmentalization, geometry, cell density, chemical‐physical properties, mechanical stimuli, etc.) that allow the recapitulation of major organ‐level functions. Based on the same concepts, tumor‐on‐chip (ToC) models have been established,^[^ ^2^, ^3^, ^4^ ^]^ where specific features of the human tumor microenvironments (TME) can be recreated with an unprecedented control. This emerging ToC technology has a huge potential for cancer research, therapy developments, drug efficacy testing, and precision medicine, providing a real solution to the ethical issues of animal experimentation.^[^ ^5^ ^]^

Endothelial cells (ECs), which form the inner lining of the blood vessels, are key components of the TME with important immunomodulatory functions. The tumor endothelium is a physical barrier that controls the recruitment of immune cells to the tumor bed. Immune recruitment is a multistep and active process finely orchestrated by physical cell–cell interactions between immune cells and ECs, as well as by complex cytokines/chemokines signaling interplay between immune, endothelial, and tumor compartments.^[^ ^6^ ^]^ Aside from its major role in promoting immune cell recruitment into tissues upon inflammation, endothelial cells can directly promote immunosuppression by down‐regulating the expression of adhesion molecules (such as E‐selectin, VCAM‐1, ICAMs, VE‐Cadherin) and by releasing specific immunomodulatory cytokines (such as IL6,^[^ ^7^ ^]^ CXCL9, CXCL10, or CXCL11^[^ ^8^ ^]^). Furthermore, endothelial cells might express molecules that affect immune cell survival, function, and phenotype (such as the death ligand FAS‐L, the immunosuppressive enzyme IDO‐1 or even immune checkpoints, such as PDL‐1/2) and by other mechanisms that have been suggested by the recent molecular elucidation of EC heterogeneity^[^ ^9^ ^]^ but that remain to be deciphered by functional assays. Since these immunosuppressive effects of EC may lead to resistance to immunotherapies,^[^ ^10^, ^11^ ^]^ combinations of anti‐tumor strategies targeting the vascular compartment and immune checkpoint blockade are currently under investigation in many clinical trials, with few combinations being recently approved by Food and Drug Administration.^[^ ^12^, ^13^ ^]^

The establishment of robust in vitro models is pivotal to further study endothelium immunomodulatory functions. Several approaches have been implemented to achieve the vascularization of tumor‐on‐chips. The first simplest vascularized ToC (vToC) models recreated tumor‐vascular interfaces inside 3D micro‐channels, using micro‐pillars^[^ ^14^ ^]^ or porous membranes.^[^ ^15^ ^]^ However, the square‐shaped geometries and sizes of these chip designs were quite far from the in vivo situations. A very elegant approach to generate in vitro more physiological vessels exploits the natural self‐assembly capacity of ECs to form perfusable microvascular networks.^[^ ^16^ ^]^ However, EC self‐organization occurs only in fibrin matrix, not representative of a tumor extracellular matrix, and additionally it is not possible to control the 3D organization of the resulting microvascular network. A compromise solution to microfabricate vessels on chip is the use of guiding needles as sacrificial molds.^[^ ^17^, ^18^, ^19^, ^20^, ^21^, ^22^ ^]^ Although this approach allows to produce simple linear microchannels without complex tortuosity, it offers great advantages in terms of precise control of vessel round‐shape and diameter, cell seeding, lumen perfusion, and 3D matrix composition. In this study, we choose this technological approach to generate our vToC.

One of the most appealing ambitions in ToC field is the development of reliable models for personalized medicine, in order to predict treatment efficacy for cancer patients and to contribute to the clinical decision‐making process. However, the current vToC models still lack several crucial properties to be clinically relevant, especially the right endothelial cell origin (i.e., microvascular cells of the organ of interest), the appropriate cellular microenvironment (i.e., co‐culture with autologous cells derived from the same patient), and the personalization (i.e., ECs specifically derived from each patient). In fact, a major limitation is the well‐known difficulty to work with primary ECs. Human umbilical vein endothelial cells (HUVEC) are the most used source of ECs by far. According to a recent comprehensive review of ToC literature, about 75% of publications concerning vToC utilize HUVEC as endothelial cell model.^[^ ^5^ ^]^ However, since HUVEC are derived from the endothelium of veins from the umbilical cord, they are macrovascular cells and they cannot be considered a good model for the microvascular ECs of the TME. Microvascular ECs are difficult to isolate and culture, and very costly when commercially available.

Here, we describe a vessel on‐a‐chip platform where a single and perfusable endothelial microvessel is generated within a collagen extracellular matrix, using patient‐derived microvascular ECs that are freshly isolated from surgical lung cancer samples. Lung cancer cells (immortalized or primary), cancer‐associated fibroblasts (CAFs), and CD8+ tumor‐infiltrating lymphocytes (TILs) are embedded in the collagen matrix to partially recapitulate the lung TME. We exploited this unique patient‐derived vascularized lung‐tumor‐on‐chip to investigate the immunomodulatory functions of the endothelium using a transcriptomic approach. Thanks to the controllability of the 3D co‐culture conditions, we were able to decipher how the TME cellular composition affects the differential expression of specific immunomodulatory genes by ECs. Finally, we provided the proof‐of‐concept of feasibility to generate personalized immunocompetent vToC composed of four autologous cell types, which is a major technological and conceptual milestone toward clinically relevant tumor‐on‐chip models.

## 2 Results

### 2.1 Implementation of Patient‐Derived Vascularized Lung Tumor‐on‐Chip

**Figure 1:**
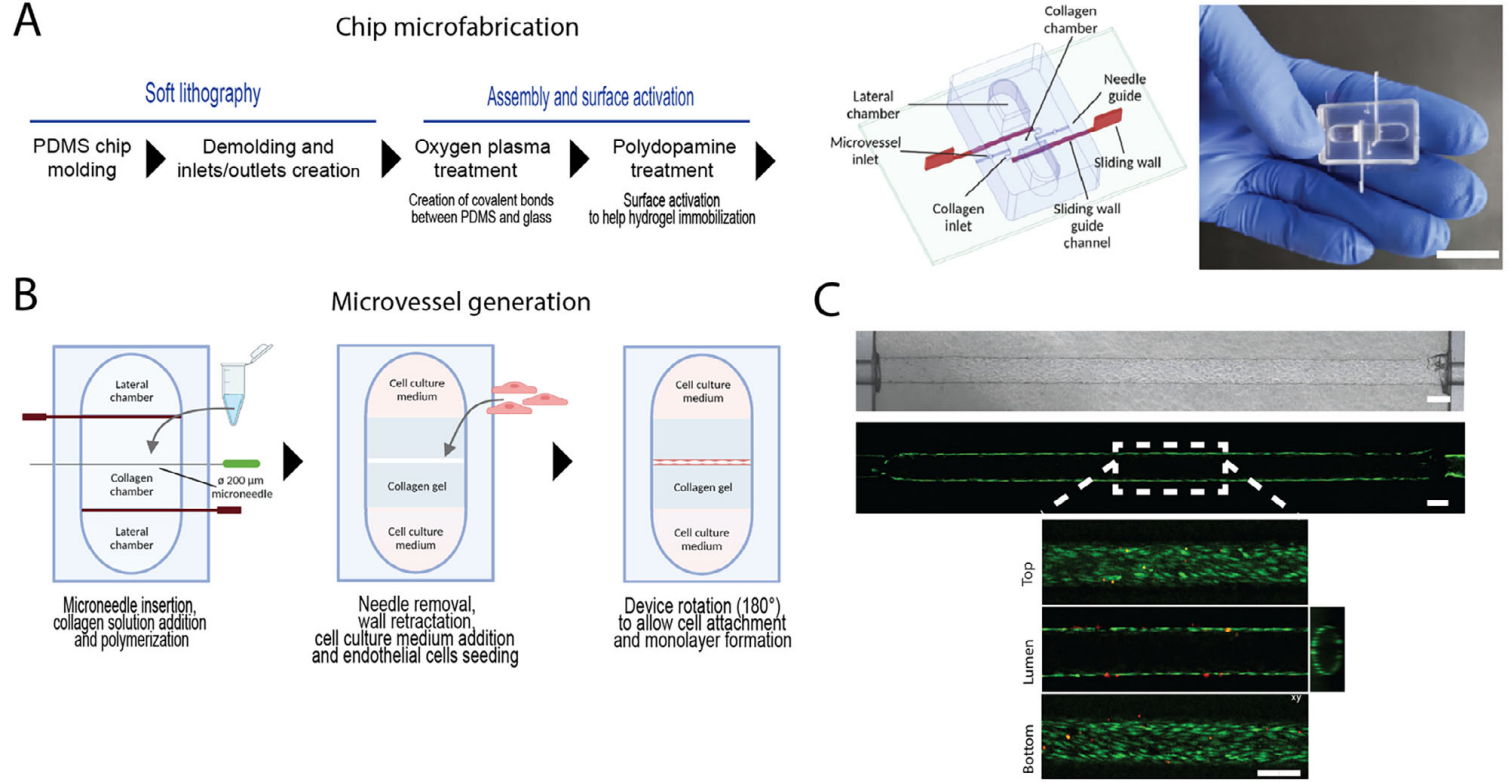
In vitro bioengineering of lung microvessels. A) Overview of the PDMS microfluidic chip fabrication. Scale bar = 1 cm. B) Overview of the microvessel generation. C) Representative phase contrast image of the full‐length microvessel (upper picture) and fluorescence images (lower picture) showing the live (green) and dead (red) cells after 48 h culture. Commercial lung endothelial cells (ECs) were used. Scale bars = 200 µm.

For in vitro bioengineering of microvessels, we conceived a novel chip design that combines two microfabrication aspects: the needle‐molding technique to generate a tubular opening within a 3D matrix of collagen type I^[^ ^17^, ^18^, ^19^, ^20^, ^21^, ^22^ ^]^ and the sliding walls to confine the collagen gel^[^ ^23^ ^]^ (**Figures** **1**A and S1, Supporting Information). The choice of sliding walls overcame many limitations of the other main approaches that enable the control of hydrogel positioning on‐chip, such as laminar flow‐based or capillary‐based approaches. In particular, the sliding walls offered an obstacle‐free interface while not being sensitive to the viscosity of the pre‐polymer hydrogel, with no limitation in chip height, allowing to easily accommodate relatively big structures such as microvessels of few hundred microns within their surrounding environment.

The microfluidic device comprised two lateral channels for cell culture medium and a central chamber containing a collagen I hydrogel. Two sliding walls were kept on closed position to allow confinement of the collagen solution and were retracted after polymerization to allow medium diffusion into the collagen gel and to obtain an obstacle‐free interface between the gel and the medium. In order to create the on chip‐vessel, a needle of 200 µm in diameter was introduced before filling the central chamber with the prepolymer solution of collagen type I (Figure 1B). Once the collagen polymerized, the needle was removed, resulting in hollow collagen channel that seamlessly connected with the polydimethylsiloxane (PDMS) channels of the chip. After coating with fibronectin, endothelial cells were seeded within this hollow channel. To implement the microvessel model we first used commercial human primary microvascular ECs from lung (HMVEC‐L). Device rotation allowed endothelial cell adhesion on all sides of the microchannel. After 24–48 h of culture, a confluent endothelial monolayer was formed along the microchannel within the collagen chamber. The engineered microvessels had a complete cell coverage along their total length of 3.75 mm and a circular hollow cross‐section with average diameter of 209 ± 1.4 µm (*n* = 47). Live/dead staining showed 92.5 ± 2% cell viability (*n* = 3) (Figure 1C). This microvessel model was viable and stable for at least 3 days without flow. The chips were perfusable, but we did not apply flow for most experiments of this manuscript.

**Figure 2:**
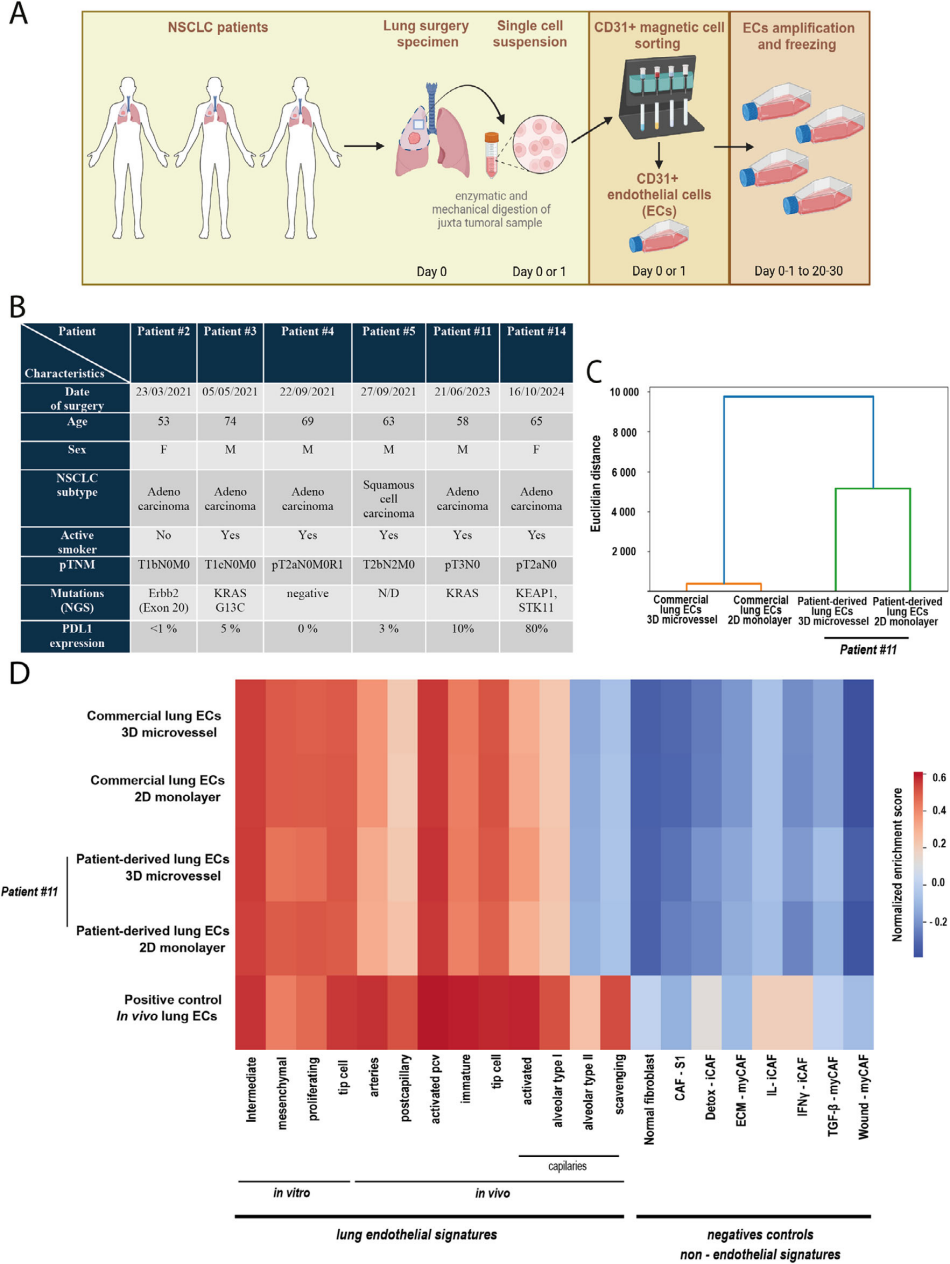
Patient‐derived lung endothelial cells isolation and transcriptomic characterization. A) Overview of isolation of endothelial cells (ECs) from juxtatumoral tissue of surgical NSCLC samples. B) Patients’ clinical data. C) Hierarchical clustering of bulk RNA‐Seq data of commercial (HMVEC‐L) and patient‐derived (patient #11) lung ECs, in standard 2D monolayer culture or 3D microvessel model (*n* = 1 sample per condition). D) Geneset enrichment scores (ssGSEA), computed from bulk RNA‐Seq data of commercial and patient‐derived (patient #11) lung ECs, in standard 2D monolayer culture or 3D microvessel model, are shown as heatmap (*n* = 1 sample per condition). Rows indicate datasets. Columns indicate signatures. As positive dataset control, the pseudo‐bulk data from an in vivo lung endothelial single‐cell RNA‐Seq atlas were used.^[^ ^9^ ^]^ The first 13 columns correspond to published lung endothelial signatures (four in vitro signatures, nine in vivo signatures) as described in.^[^ ^9^ ^]^ As non‐endothelial negative control signatures, fibroblast signatures were used: one normal fibroblast signature and seven cancer‐associated fibroblast (CAF) signatures (CAF‐S1 subtypes).

We next developed a strategy to freshly isolate microvascular endothelial cells from surgical samples of non‐small cell lung cancer (NSCLC) patients. Our attempts to isolate viable endothelial cells (ECs) from the tumor tissue failed because of the very low number of tumor ECs. As alternative, since lung surgery consists in removal of the entire lobe or segment, we succeeded in efficient EC isolation starting from the healthy lung portion (sample weight about 2–5g). The EC isolation procedure involves sequential magnetic‐activated cell sorting (MACS) using anti‐CD31 conjugated microbeads followed by culturing cells in appropriate endothelium medium on gelatin‐coated flasks (**Figure** **2**A). With this protocol, we isolated and cultured ECs with 100% success rate from six patients (Figure 2B, Figure S2, Supporting Information). 4–5 days after CD31 MACS, the observed adherent cells started to form clusters and cells reached confluence after 6 to 10 days. At this point, the cells showed the typical EC‐like cobblestone morphology under observation with a phase‐contrast microscope (Figure S3A, Supporting Information). Isolated ECs were kept in culture and amplified for up to 20 days, with additional one or two rounds of CD31 MACS selections, depending on the residual presence or not of contaminant cells, mainly CAFs, that can be visually recognized based on their typical fibroblastic morphology. Low‐passage cells were frozen for further use.

Endothelial identity was ascertained by immunofluorescence microscopy, flow cytometry, and transcriptomic analysis. Commercial lung ECs (HMVEC‐L) and fibroblasts were used as positive and negative controls, respectively. Immunofluorescence images showed a homogeneous population for the freshly isolated ECs, with positive staining for two EC markers: VE‐cadherin at the cell–cell junctions and intracellular Willebrand factor (vWF) (Figure S3B, Supporting Information). The >98% purity of isolated ECs was confirmed by flow cytometry using the CD31 endothelial marker (Figure S3C, Supporting Information). Bulk RNA‐Seq analysis was performed on commercial and patient‐derived ECs, both as monolayer in gelatin‐coated 2D dishes and as endothelium in the 3D microvessels. Hierarchical clustering indicated that EC source (commercial or patient‐derived) has more impact on the transcriptional profile than the geometry (2D monolayer or 3D microvessel) (Figure 2C). We used published EC signatures (four in vitro signatures and nine in vivo signatures)^[^ ^9^ ^]^ to score our EC samples by gene set enrichment analysis (Figure 2D). Non‐endothelial signatures were used as negative controls. Our EC samples displayed a clear endothelial identity, with a strong similarity among them. As expected, the in vitro EC signatures showed higher scores as compared to in vivo EC signatures, consistently with the established notion that EC heterogeneity observed in vivo by scRNA‐Seq is largely lost when cells are cultured ex vivo.^[^ ^9^ ^]^

### 2.2 Phenotypic and Functional Characterization of Engineered Lung Microvessels

**Figure 3:**
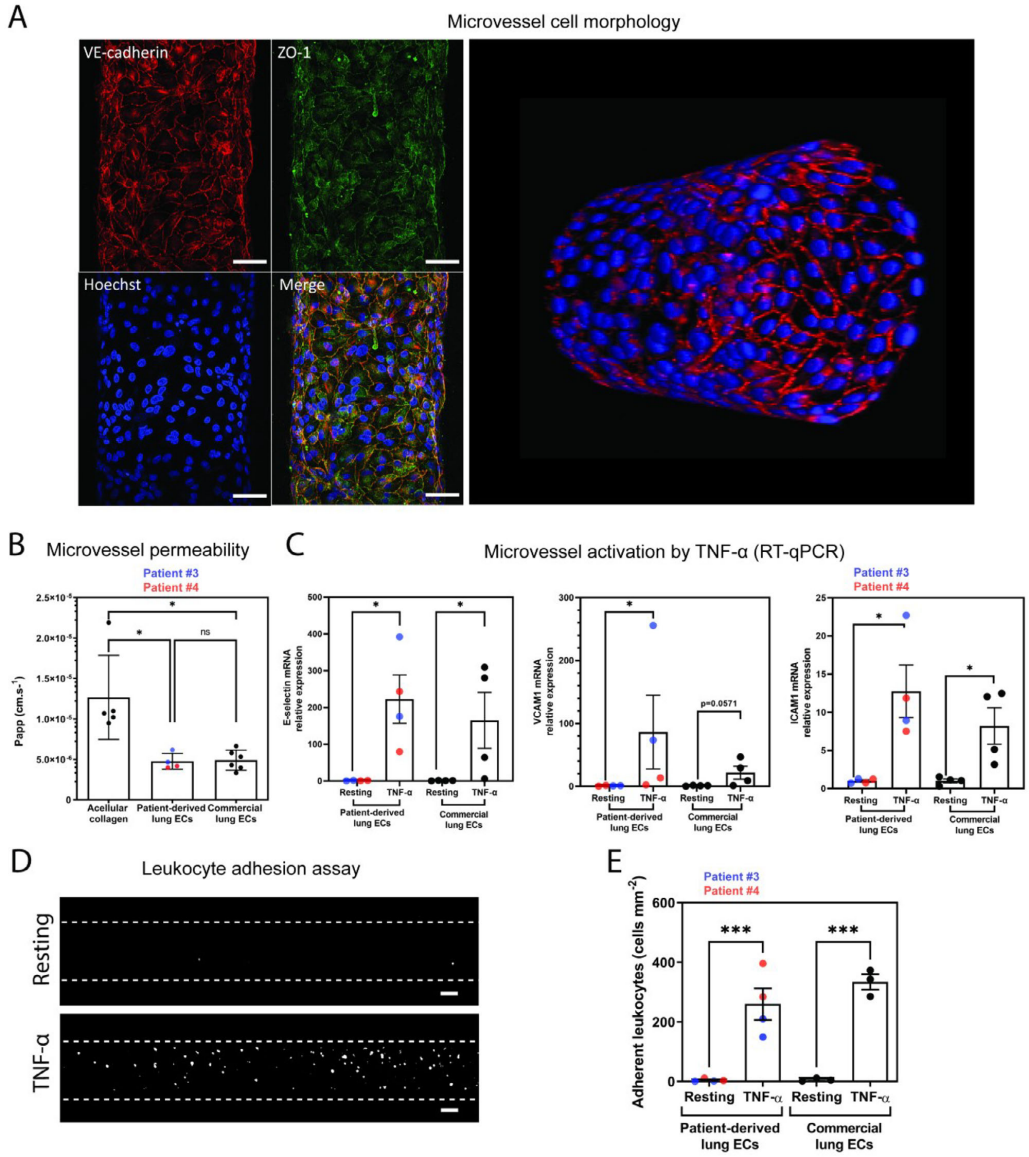
Phenotypic and functional characterization of engineered patient‐derived lung microvessels. A) Confocal imaging of engineered vessels. Representative images of VE‐cadherin (red), ZO‐1 (green), and Hoechst 33342 (blue) staining on a vessel generated with patient‐derived ECs (patient #4) after 24 h culture (left panel). Representative 3D projection (HMVEC‐L cells, right panel, see also Movie S1, Supporting Information). Scale bar = 50 µm. B) Microvessel permeability. Endothelial barrier apparent permeability (Papp) was determined by FITC‐dextran (70 kDa) permeability assay. Commercial lung endothelial HMVEC‐L cells and acellular collagen served as positive and negative controls, respectively. The mean ± standard error of the mean (SEM) are shown. Each point represents an independent microvessel, *n* = 4–6 per condition. Data distribution was assessed using the Shapiro–Wilk test and did not meet the assumption of normality; consequently, statistical analysis was conducted using the Kruskal–Wallis test followed by Dunn's multiple comparisons test. ns = not significant, * = *p* < 0.05, ** = *p* < 0.01, *** = *p* < 0.001. C) Activation by TNF‐α. mRNA levels of ICAM‐1, VCAM‐1, and E‐selectin in lung microvessels were analyzed by mRNA under resting conditions and after activation with TNF‐α (100 ng mL^−1^ for 4 h). Commercial lung endothelial HMVEC‐L cells served as positive control. The housekeeping β2‐microglobuline (B2M) gene was used to normalize RNA contents. Relative expression = 2^−∆∆Ct^. Mean 2^−∆∆Ct^ is presented ± SEM, *n* = 4 independent microvessels per condition. Data distribution was assessed using the Shapiro–Wilk test and did not meet the assumption of normality; consequently, statistical analysis was conducted using Mann–Whitney test. ns = not significant, * = *p* < 0.05, ** = *p* < 0.01, *** = *p* < 0.001. D) Leucocyte adhesion assays. Adhesion assays were carried out by perfusing fluorescently labelled Jurkat T lymphocytes on resting or TNF‐α treated lung microvessels. Scale bars = 50 µm. E) Quantifications of adhesion assays. Number of firmly adherent T‐lymphocytes was quantified per area. The mean ± SEM are shown, *n* = 3–4 independent microvessels per condition. Commercial lung endothelial HMVEC‐L cells served as positive control. Data distribution was assessed using the Shapiro–Wilk test and met the assumption of normality; consequently, statistical analysis was conducted using one‐way ANOVA Tukey's multiple comparisons test. ns = not significant, * = *p* < 0.05, ** = *p* < 0.01, *** = *p* < 0.001.

The integrity of the endothelial monolayer along the whole inner surface of the collagen tube was assessed by immunofluorescence and confocal microscopy. The staining of the *adherens* junction protein VE‐cadherin and the tight junction‐associated protein ZO‐1 showed that endothelial cells formed a continuous compact monolayer with proper cell–cell junctions, already as soon as 24 h post‐cell seeding (**Figure** **3**A; Movie S1, Supporting Information). To functionally assess the formation of an endothelial barrier, the vessel permeability was quantified by the permeability assay using fluorescent dextran (70 kDa). The diffusion of fluorescent‐dextran across the endothelium was measured after its continuous perfusion through the vessel and by recording fluorescent images every 10 s. The results indicated that the presence of the endothelial cells, either freshly isolated from surgical samples of two patients or commercial HMVEC‐L, similarly limited the diffusion of the fluorescent macromolecule across the endothelium, compared to acellular collagen tubes (Figure 3B; Figure S4, Supporting Information). The apparent permeability was significantly reduced in channels covered by endothelial cells (apparent permeability, *Papp*, 4.9 × 10^−6^ ± 1.24 × 10^−6^ cm s^−1^ for HMVEC‐L, 4.75 × 10^−6^ ± 0.99 × 10^−6^ cm s^−1^ for patient ECs) with respect to acellular channels (*Papp*, 1.25 × 10^−5^ ± 0.52*10^−5^ cm s^−1^), demonstrating the formation of an efficient barrier function after 24 h post‐cell seeding.

In addition, we evaluated endothelium functionality by assessing its quiescence as well as its ability to switch from a quiescent to an immune activated state. At quiescent or resting state, the ECs do not bind leukocytes. Under stimulation with pro‐inflammatory cytokines, such as TNF‐α, the expression of surface adhesion molecules is upregulated on the endothelium, promoting leukocyte adhesion.^[^ ^24^ ^]^ Real‐time quantitative PCR (RT‐qPCR) analysis showed that the mRNA levels of the adhesion molecules ICAM‐1, VCAM‐1, and E‐selectin were highly upregulated upon TNF‐α treatment (100 ng mL^−1^, for 4 h) in lung microvessels formed by both patient‐derived or commercial ECs, already after 24 h post‐cell seeding (Figure 3C). Additionally, upon TNF‐α treatment, the number of adherent leukocytes on the microvessels was significantly higher compared to resting conditions (Figure 3D,E). All together these data validated the functional state of our engineered lung microvessels generated from patients’ samples.

### 2.3 Transcriptomic Approach in vToC to Assess Immunomodulation by the Endothelium

**Figure 4:**
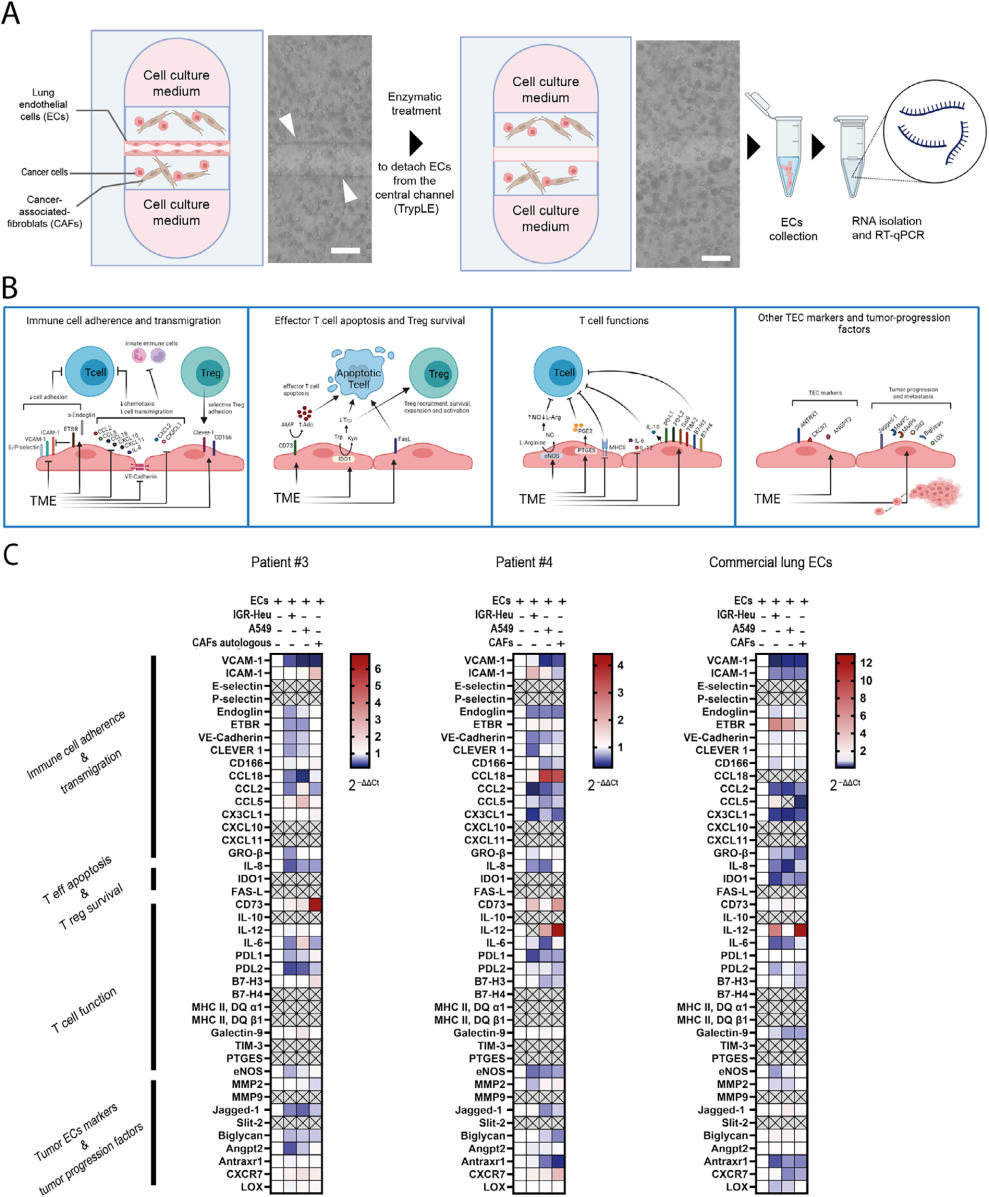
Impact of TME cell components on endothelium‐mediated immunomodulation. A) Isolation of endothelial RNA from vascularized tumor‐on‐chip. On‐chip microvessels are cultured alone or with other TME cells (cancer cells, CAFs) embedded in the collagen gel. ECs are specifically retrieved from the chip using TrypLE enzyme, followed by RNA extraction and RT‐qPCR analysis. Microscopy pictures before (left) and after TrypLE dissociation (right) show the specific removal of endothelial cells. White arrows are pointing ECs monolayer. Scale bars = 200 µm. B) Description of the gene panel. 42 endothelial genes were selected for their reported contributions to immunomodulation and tumor progression. C) Analysis of 42‐gene panel expression in microvessels co‐cultured with cancer cells or CAFs. RT‐qPCR analysis was done on patient‐derived ECs (patients #3 and #4), or commercial HMVEC‐L, after 24 h co‐culture. Two cell lines, A549 and IGR‐Heu, were used as lung cancer cell models, and either autologous CAFs (patient #3) or commercial CAF07AD were used as CAF model. mRNA relative expression (2^−ΔΔ^ *^C^* ^t^) is reported relatively to control condition (ECs alone), *n* = 1–3 independent microvessels. Gray crossed boxes represent condition where the gene expression was undetected in RT‐qPCR using TLDA (Taqman Low density array) technique.

With the aim to specifically and efficiently investigate the regulation of endothelial genes in the context of the in vitro reconstructed TME, we conceived an experimental strategy to isolate the RNA from the ECs lining the microvessels, without any RNA contamination from the non‐endothelial cells embedded in the collagen. ECs were dissociated with TryPLE enzyme which does not digest the collagen, then collected and immediately lysed in TRIzol (**Figure** **4**A). Despite the low number of EC cells (≈1500 cells per microvessel), the amount of the extracted RNA was sufficient for subsequent RT‐qPCR analysis. Importantly, the fact that we did not detect any mRNA of cancer‐specific genes (AZGP1 and KRT19), nor of CAF‐specific genes (LUM and DCN), confirmed the absence of non‐endothelial RNA contamination. In contrary, we clearly detected mRNA of endothelial‐specific genes (PLVAP and vWF), validating the RNA extraction method (Figure S5, Supporting Information).

Next, based on an extensive literature search on the interplay between endothelium and immunity, we rationally constructed a panel of 42 genes (with six additional housekeeping genes) which are expressed by ECs and known to be involved in immunomodulation, more specifically in immune cell adhesion, transmigration, effector T cell apoptosis, Treg survival, and T cell functions (Figure 4B; Figure S6, Supporting Information). We also included to the panel genes involved in tumor progression and metastasis spreading or genes reported to be marker of tumor endothelial cells (TEC), reasoning that immune infiltration and cancer cell extravasation share common molecular mechanisms.

In more details, our panel comprises genes coding for proteins directly involved in immune recruitment, such as adhesion molecules (E‐and P‐selectin, VCAM‐1, ICAM‐1),^[^ ^25^, ^26^, ^27^ ^]^ regulators of cell adhesion molecules (endothelin receptor B^[^ ^28^ ^]^ and endoglin^[^ ^29^, ^30^ ^]^), as well as a large panel of chemokines and cytokines regulating immune infiltration (CCL2,^[^ ^31^ ^]^ CCL5,^[^ ^32^ ^]^ CCL18, CXCL10, CXCL11,^[^ ^33^ ^]^ IL‐8,^[^ ^34^ ^]^ CXCL2, and CX3CL1) and two actors reported to promote selective adhesion of regulatory T cells (CLEVER^[^ ^35^ ^]^ and CD166). Among regulators of T cell apoptosis/survival, we chose CD73 (an ecto‐nucleotidase that catalyzes the hydrolysis of extracellular adenosine monophosphate into adenosine which is a powerful pro‐apoptotic signal for CD8+ T‐cells),^[^ ^36^ ^]^ IDO1 (an enzyme involved in metabolism of tryptophan that promotes CD8+ T‐cell activity),^[^ ^37^ ^]^ and the death ligand FAS L (which induces FAS dependent apoptosis).^[^ ^38^ ^]^ Selected immune check points ligands (PD‐L1,^[^ ^39^ ^]^ PD‐L2,^[^ ^40^ ^]^ TIM‐3,^[^ ^41^ ^]^ B7‐H3/CD276,^[^ ^42^, ^43^ ^]^ B7‐H4/VTCN1^[^ ^44^ ^]^), interleukines (IL‐6^[^ ^45^ ^]^ and IL‐12), anti‐inflammatory molecules (eNOS,^[^ ^46^ ^]^ PTGES,^[^ ^47^ ^]^ and galectin 9^[^ ^11^ ^]^), and major histocompatibility complex (MHC) Class II proteins (HLA DQ α1 and β1^[^ ^48^ ^]^) were also added since they have been reported to be expressed by ECs and could promote an immunosuppressive ecosystem. We selected CXCR7,^[^ ^49^ ^]^ angiopoietin 2,^[^ ^50^ ^]^ and ANTXR1 (also known as tumor endothelial marker 8, TEM 8),^[^ ^51^ ^]^ as putative markers of TEC. We added six actors having a role in extracellular matrix (ECM) remodeling, thus promoting angiogenesis, tumor cell migration, and metastatic spreading: two members of the matrix metalloprotease family (MMP2 and MMP9),^[^ ^52^ ^]^ the copper‐dependent amine oxidase LOX,^[^ ^53^ ^]^ the proteoglycan biglycan,^[^ ^54^ ^]^ Slit2, a secreted protein with potential of cancer cell guidance,^[^ ^55^ ^]^ and Jagged1, a notch ligand.^[^ ^56^ ^]^

By RT‐qPCR analysis using custom‐made Taqman arrays, we first applied this gene panel to microvessels made of patient‐derived cells with or without TNF‐α treatment (Figure S7A, Supporting Information). As expected, we observed again the strong upregulation of genes encoding for adhesion molecules: ICAM‐1, VCAM‐1, and E‐selectin as above, and more mildly also of P‐selectin. Actually, most of the genes of the panel reported to be associated to immune effective response (CCL18, CCL2, CCL5, CXC3CL1, CXCL11, GROβ, IL‐8, IL‐6, and MHC II) were robustly upregulated, confirming that the endothelium of lung microvessels was in resting state and did not exhibit a basal endothelial anergy, since we observed a stereotypical response to pro‐inflammatory stimuli. Few immunosuppressive actors were also down regulated (endoglin, CD166, CD73, PDL2, and eNOS), reinforcing endothelial activation and pro‐inflammatory state upon TNF‐α stimulation.

Next, we compared the endothelium of 3D patient‐derived microvessels with the same ECs grown in gelatin‐coated 2D dishes (Figure S7B, Supporting Information), in resting state without TNF‐α. Interestingly, ECs cells cultured in 2D dishes showed increased expression of several adhesion molecules (ICAM‐1, VCAM‐1, E‐selectin, and P‐), as well as of several attractant chemokines (CCL18, CCL2, CCL5, and CX3CL1) and of molecules of the MHC (MHC class II DQ α1 and β1). This suggests that 2D culture conditions may lead to partial and reversible, not physiological, endothelium activation and reinforcing the importance to mimic the in vivo 3D settings, including geometry and stiffness.

### 2.4 Impact of TME Cell Components on Endothelium‐Mediated Immunomodulation

We embedded TME cell populations in the collagen surrounding the microvessel. We first started with two immortalized human cell lines: A549 lung adenocarcinoma cells (from ATCC) and IGR‐Heu large cell carcinoma cells (established at Gustave Roussy Institute).^[^ ^57^ ^]^ As lung CAF cell models, we used primary CAFs isolated from patient #3 as well as the commercial CAF07A (Neuromics). Primary CAFs easily grow on standard culture dishes. Based on the expertise of our laboratory on CAF heterogeneity,^[^ ^58^, ^59^, ^60^, ^61^, ^62^ ^]^ we know that CAFs cultured in standard 2D dishes correspond to the immunosuppressive FAP+ CAF‐S1 subpopulation, ECM‐myCAF subset.

In order to identify conserved TME modulations, independent of the specific cell models, we generated vascularized tumor‐on‐chip using three different types of lung ECs (patient #3, patient #4, HMVEC‐L), two different lung cancer cell lines (A549, IGR‐Heu), and two different primary types of lung CAF‐S1 (patient #3, commercial). After 24 h of on‐chip co‐culture and endothelium formation, ECs’ RNA were extracted and RT‐qPCR array analysis performed using the 42‐gene panel previously described. The Δ*C*_t_ values of the 42 genes were very consistent between the three endothelial cell types (Figure S8, Supporting Information).

The panel gene expression profiles of the endothelium in the various co‐culture conditions were reported relatively to the control condition with only the endothelium (Figure 4C). The expression of about one third of the genes was too low to be detected using TLDA (Taqman low density array). Despite variability depending on the cell models, overall, the presence of cancer cells or CAFs had mostly a negative impact on immunomodulatory gene expression rather than a positive effect (on average 64.5% ± 6.5% of the detected genes were down regulated, i.e., fold change <1 with respect to ECs alone condition). There were some interesting exceptions. For example, CD73, the ecto‐nucleotidase responsible for production of extra‐cellular adenosine which is a potent immunosuppressor^[^ ^36^ ^]^ and which appeared to be upregulated in the presence of cancer cells and CAFs in the two patient‐derived microvessels. For patient #3 and patient #4, mean CD73 mRNA expression levels compared to ECs alone condition, were increased upon co‐cultures (1.40, 1.71, and 6.87 for patient #3, and 1.8, 1.09, and 2.36, in patient #4, with IGR‐Heu, A549 and CAFs, respectively). However, no substantial modulation was observed with HMVEC‐L cells (0.97, 1.71, and 0.98‐fold changes upon co‐culture with IGR‐Heu, A549 and CAFs, respectively).

**Figure 5:**
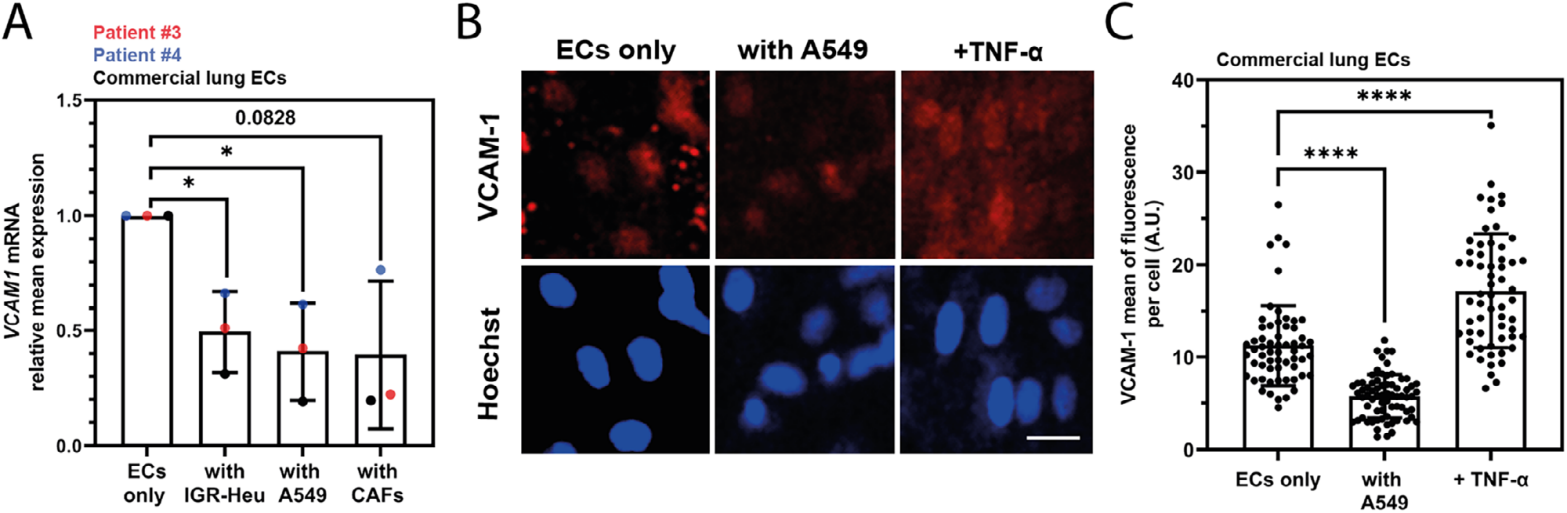
VCAM‐1 down‐regulation in lung microvessels. A) RT‐qPCR analysis of *VCAM1* mRNA expression in lung microvessels co‐cultured for 24 h with cancer cells (IGR‐Heu or A549) or CAFs. Each dot represents the average value of VCAM‐1 mRNA relative expression from *n* = 1‐3 independent microvessels per condition. The mean ± standard deviation is shown. Statistical Shapiro normality test and paired T‐test were used. ns = not significant, * = *p* < 0.05, ** = *p* < 0.01, *** = *p* < 0.001. B) Representative VCAM‐1 immunostaining images of lung microvessels with ECs only (commercial HMVEC‐L), co‐cultured for 24 h with A549 cancer cells, or stimulated with TNF‐α (100 ng mL^−1^ for 4 h). Hoechst was used for nuclear staining. Scale bar = 20 µm. C) Quantification of mean fluorescence staining of VCAM‐1 protein per cell. *n* = 60–80 cells were manually quantified from two independent chips per condition. The mean ± standard deviation is shown. Since Statistical Shapiro normality test was not passed, Kruskal–Wallis test followed by Dunn's multiple comparisons test was used. ns = not significant, * = *p* < 0.05, ** = *p* < 0.01, *** = *p* < 0.001.

Interestingly, VCAM‐1 expression was systematically and robustly downregulated in all three endothelial cell models and in all three co‐cultures conditions (0.44, 0.16, and 0.17‐fold changes, compared to ECs alone condition, for patient #3, upon co‐culture with IGR‐Heu, A549 and CAFs, respectively; 0.95, 0.37, and 0.49‐fold changes for patient #4; 0.10, 0.23, and‐0.24 fold changes for HMVEC‐L). The results of this array analysis on VCAM‐1 expression were confirmed by standard duplex RT‐qPCR analysis (**Figure** **5**A). Moreover, a substantial down‐regulation of VCAM‐1 within the microvessel was validated at protein level by immunostaining: upon co‐culture with A549 cancer cells, VCAM‐1 protein level in ECs decreased of two folds (Figure 5B,C).

Another interesting modulation is the overall robust down‐regulation of CCL2 chemokine in the three endothelial cell models, in eight out nine co‐culture conditions with cancer cells or CAFs (Figure 4C). Since CCL2^[^ ^31^ ^]^ is a major player in chemoattraction of immune cells, particularly monocytes, this finding suggests that the presence of cancer cells and CAFs in TME might impair CCL2 production by endothelium and decrease immune recruitment.

### 2.5 Generation of a Patient‐Derived Immunocompetent Vascularized Lung Tumor‐on‐Chip

**Figure 6:**
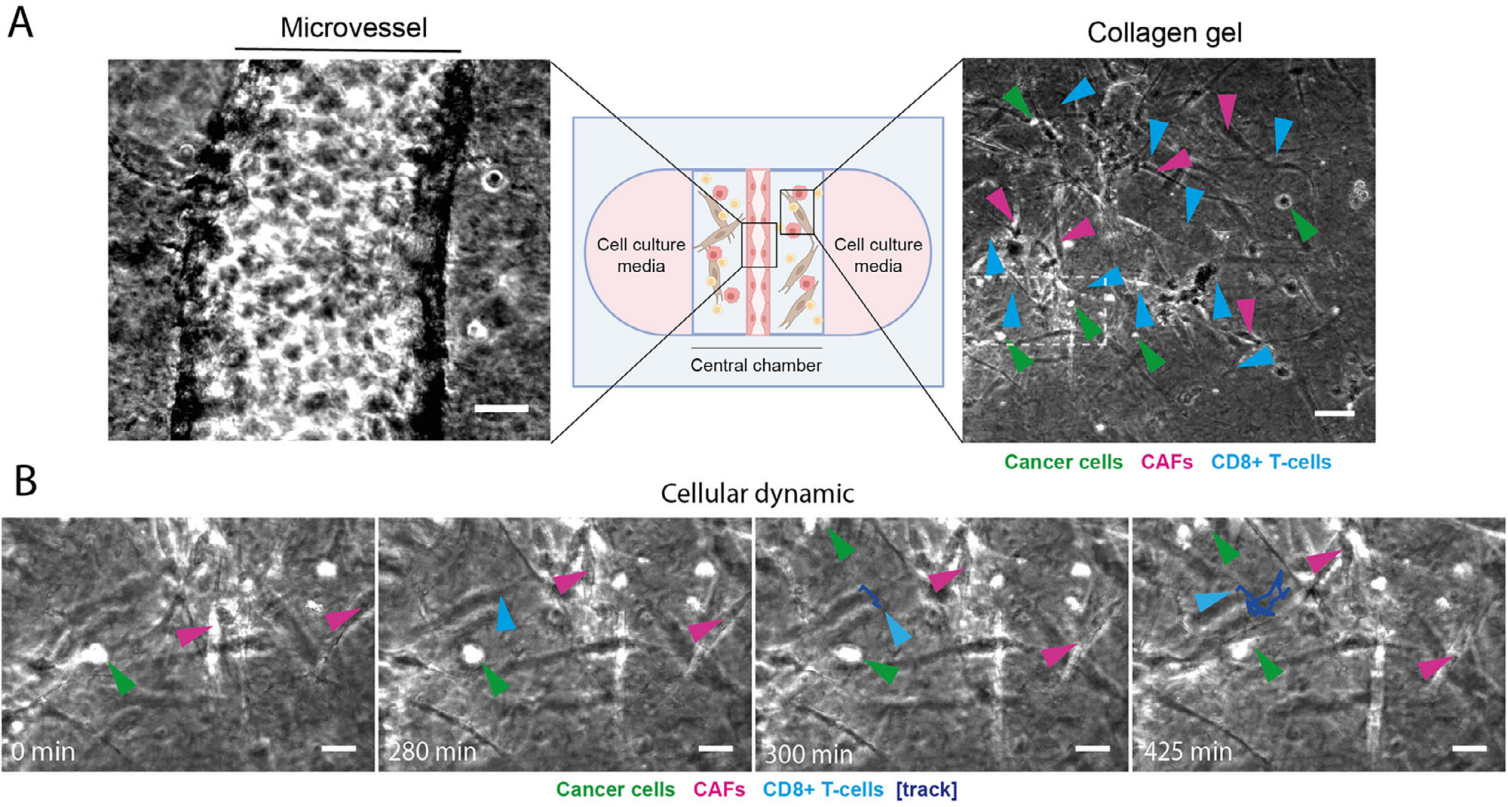
Generation of a patient‐derived vascularized lung tumor‐on‐chip with four primary autologous cell populations (ECs, cancer cells, CAFs, CD8+ T‐cells). A) Representative transmitted light microscopy images of the vToC from patient #14 showing the endothelial microvessel (left, see also Movie S2, Supporting Information) and collagen‐embedded autologous lung cancer cells (green arrows), CAFs (pink arrows), and CD8+ T‐cells (light blue arrows) (right, see also Movie S3, Supporting Information). The white dashed area corresponds to the region of interest (ROI) used for panel B. Scale bars = 50 µm. B) Time‐lapse images showing the cellular dynamic within the ROI depicted in panel A (see also Movie S4, Supporting Information). Lung cancer cells (green arrows), CAFs (pink arrows), and CD8+ T‐cells (light blue arrows) are pointed at different time points (0, 280, 300, and 425 min). The track of one CD8+ T‐cell is drawn in dark blue. Scale bars = 20 µm.

In order to generate a fully autologous vToC, with tumor‐infiltrating immune cells, we processed the surgical sample of patient #14 by combining the EC isolation protocol from healthy tissue as described above (Figure 2A) with the isolation protocol of cancer cells, CAFs and CD8+ TILs from tumor tissue, which we previously described.^[^ ^63^ ^]^ The four autologous cell populations were cultured in appropriate media and supports for few weeks (see Experimental Section) before seeding in the vToC. Live cell imaging started after ECs adhesion, for 48 h observation time (**Figure** **6**; Movies S2–S4, Supporting Information). All the four cell types displayed the expected morphologies and dynamics. The ECs within the microvessel endothelium appeared compact and active. Cancer cells were round and static, with dynamic plasma‐membrane movements. CAFs were very elongated, with long protrusions and slow‐moving. CD8+ T‐cells were moving very fast and engaging many cell–cell contacts. By applying a sufficiently high acquisition frequency (1 image every 1–5 min), T‐cell trajectories were manually tracked (Figure 6B). Overall, these observations demonstrated for the first time the feasibility to reconstitute patient‐derived tumor ecosystem, composed of cancer cells and fully autologous CAFs, ECs, and immune cells.

## 3 Discussion

The main aim of this study was to establish a clinically relevant in vitro model of vascularized TME by using patient‐derived ECs. Our initial ambition was to isolate ECs directly from the lung tumor tissues, at the same time as primary cancer cells, TILs and CAFs,^[^ ^63^ ^]^ but the amount of extracted tumor ECs was not sufficient to establish primary culture, likely because ECs represent only a very low fraction (≈2%) of the overall cells in tumors.^[^ ^64^ ^]^ However, we succeeded in obtaining juxta tumoral ECs (≥99% purity) from healthy portions of patients’ lung lobes removed during surgery (100% success rate, six out six patients). Other studies showed the feasibility of establishing primary culture of tumoral ECs, in addition to juxta tumoral ECs,^[^ ^9^, ^65^ ^]^ but without the possibility to simultaneously isolate other TME cell types.

We compared microvessels generated with patient‐derived ECs with microvessels generated with commercial lung primary microvascular ECs from healthy donors (HMVEC‐L). Overall, the phenotypic, functional, and transcriptomic analyses showed similar behaviors, indicating that our EC isolation/amplification protocols, and chip fabrication methodology, allow to efficiently generate functional microvessels with the expected endothelium properties. In this work we used a very small patients’ cohort (six patients), therefore we could not statistically evaluate inter‐patient differences. However, the personalization of our vToC with patient‐derived ECs opens the possibility to address new questions, such the impact of patient variability on endothelium properties, response to drugs, or interplay with other TME components. For renal cell carcinoma, in vitro microvessel models were generated with patient‐derived ECs and used for treatment testing, such as anti‐angiogenic drugs,^[^ ^65^, ^66^ ^]^ supporting the notion that vToC models have potential applications to inform on patient‐specific drug responses. However, appropriate clinical trials will need to be conceived to evaluate the correlation with the clinical responses of patients.

Comparing to previous tumor‐on‐chip studies,^[^ ^5^ ^]^ our experimental strategy has an important advantage in that it offers the possibility to simultaneously isolate from the same surgical sample the four primary cell populations (cancer cells, TILs, CAFs, and ECs) and to incorporate them inside the chips after short amplification. Therefore, our approach represents a significant advancement in the field by implementing a 3D perfused model using patient‐derived cells. Earlier attempts at reconstituting patient‐derived lung cancer models were mostly done using tumor organoids, which resulted in structures mainly composed of epithelial and fibroblast cells.^[^ ^67^, ^68^, ^69^, ^70^ ^]^ These tumoroids did not reconstitute a complete tumor cellular microenvironment and lacked essential components such as the perfused vasculature and the immune cell compartment. A more recent approach involved droplet microfluidics to reconstitute patient‐derived tumoroids composed of lung tumor cells, CAFs, and immune cells^[^ ^71^ ^]^ but did not reconstitute a 3D vascularized environment of the tumors. Earlier attempts at reconstituting lung tumor‐on‐chip organized in a 3D setting using microfabrication mostly used established cell lines.^[^ ^72^, ^73^ ^]^ More recently, vascularized 3D models of lung tumor on‐chip were also developed, using either reconstituted tumor spheroids,^[^ ^74^ ^]^ cells on nanofiber membrane scaffolds,^[^ ^75^ ^]^ or a more elegant 3D model reconstituting the air–epithelial interface.^[^ ^15^ ^]^ However, all these approaches which reconstituted a 3D architecture used established or primary cell lines which were not derived from lung cancer patients.

In addition, we provide here the proof‐of‐concept generation of a fully autologous vToC which is an essential step toward tumor‐on‐chip models with real clinical relevance. In particular, the autologous nature of immune T‐cells is absolutely required to avoid any allogeneic reaction, since the ECs are able to present antigen through MHC class II molecules.^[^ ^76^ ^]^ Heterologous co‐cultures of TME cells, without immune cells or with only innate immune cells, will remain useful for certain types of investigation, but only autologous ToC are appropriate to study cancer‐immune interplay and immunotherapy responses.

In this work we exploited the vToC model to address the question of the impact of TME cell components on the expression of immunomodulatory genes by the endothelium. We found that cancer cells and CAFs decreased expression by ECs of VCAM‐1 leukocyte adhesion protein, which is crucial for immune infiltration. Moreover, when ECs were in co‐culture with cancer cells and CAFs, we observed a global down‐regulation in ECs of more than half of the immunomodulatory genes within our panel, including CCL2. These results are in line with the current concept that TME factors push the endothelium toward an immunosuppressive phenotype and favor blood vessel endothelial cell anergy. Indeed, inhibition of endothelial VCAM‐1 is a hallmark of endothelial anergy and was proposed to be driven in vivo by secretion of pro‐angiogenic factors within the TME.^[^ ^77^, ^78^ ^]^ In mouse models, VCAM‐1 endothelial density was found to correlate with immune infiltration and response to immunotherapy.^[^ ^79^ ^]^ More recently, Kim et al^[^ ^80^ ^]^ described in ECs a repressive epigenetic regulation of VCAM‐1, CXCL9, and CXCL10, which was dependent on the proangiogenic factor FGF2. This non permissive status of the vasculature could be reversed by inhibition of endothelial DNA methyltransferase 1, promoting CD8+ T lymphocytes recruitment and potentiating the efficacy of immune checkpoint inhibitors in breast cancer mouse models.^[^ ^80^ ^]^

A significant limitation must be pointed out. Although the vToC model effectively integrates multiple cell types and recapitulates certain complex behaviors of the tumor ecosystem, it is still very far from capturing the full complexity of the TME. For example, as previously noted, upon ex vivo culture, both ECs and CAFs lose their cellular heterogeneity, as only specific cell subtypes are able to survive and proliferate outside the human body. Moreover, while CD8+ TILs have been incorporated on chip, the TME includes a much broader array of immune cells, with macrophages being the most abundant. Further research is needed to enhance the cellular complexity of tumor‐on‐chip models while ensuring that they remain controllable.

In conclusion, the findings of this study demonstrate that our patient‐derived vToC is a robust ex vivo platform. When combined with advanced transcriptomic analyses, it provides valuable insights into the characteristics and functions of patient‐derived endothelia within the context of a reconstituted tumor ecosystem. vToC can serve as a powerful research tool to investigate TME‐dependent molecular mechanisms and to assess the effects of anti‐cancer treatments, including anti‐angiogenic drugs and immunotherapies. Finally, vToC can be personalized with primary autologous cells to study patient‐specific features and to prepare future applications in personalized medicine.

## 4 Experimental Section

### Lung Tissue Collection

Tumor and healthy (i.e., same lobe) lung tissues were obtained from lung resections of NSCLC patients treated at Bichat–Claude Bernard Hospital (AP‐HP.Nord) in Paris. Ethical approval was obtained by the Comité d'Evaluation des Protocoles de Recherche Observationnelle (number 2020–051) from the French Speaking Respiratory Medicine Society. All patients were informed on admission that tissue samples or organs obtained during diagnostic or treatment procedures may also be used for research purposes. Through appropriate institutional informed forms, they could oppose to the use of their samples and/or clinical de‐identified associated data. Collected specimens (0.5–5 g) were placed in ice cold RPMI‐1640 medium (Gibco, 21875‐034) supplemented with 10% fetal bovine serum (FBS) (Biosera, S002T2000O) and 1% penicillin/streptomycin solution (Sigma‐Aldrich, 11074440001) for transportation and processed as soon as possible the same day.

### Cell Isolation and Culture

Tissues were fragmented into small pieces (2 mm^3^) with a scalpel and enzymatically digested with a tumor dissociation kit (Miltenyi Biotec, 130‐095‐929), according to the manufacturer's instructions. ECs were positively isolated from lung healthy tissues of lung cancer patients by MACS using the CD31 MicroBead kit (Miltenyi Biotec, 130‐091‐935). Bound cells were then eluted and passed through a second separation column to reach higher purity. CD31+ cells were plated on 1.5% w/v bovine gelatin (Sigma‐Aldrich, G1393) pre‐coated dishes at a cell density between 1 and 3.10^5^ cells per cm^2^ and incubated overnight in EGM2‐MV medium (Lonza, CC‐3156 and CC‐4147) at 37 °C with 5% CO_2_. Non‐attached cells were gently washed away, and adherent ECs were kept in culture until confluence, replacing the medium every 2 or 3 days. Commercial human primary microvascular ECs from lung (HMVEC‐L) were purchased from Lonza (CC‐2527). ECs were routinely passaged at 1:3 dilution and were used for microvessel formation at low passage (max 5).

Primary lung cancer cells, CD8+ T‐cells, and CAFs were isolated from tumor lung tissue by MACS as reported.^[^ ^63^ ^]^ Primary lung cancer cells were cultured up to 2–3 months as spheroids into ultra‐low–attachment well‐plates (Corning, 3471) or flasks (Corning, 3814), in DMEM‐F12 (Gibco, 11330‐032) supplemented with 1% penicillin/streptomycin solution (Thermo‐Fisher Scientific, 15140122), 0.1× B27 supplement serum‐free (Thermo‐Fisher Scientific, 17504044), 4 µg mL^−1^ heparin (Sigma‐Aldrich, H3149‐10KU), 5 ng mL^−1^ human insulin (Sigma‐Aldrich, I9278‐5ML), 1 µg mL^−1^ hydrocortisone (Sigma‐Aldrich, H0888‐1G), 20 ng mL^−1^ EGF (Thermo‐Fisher Scientific, PHG0311), 20 ng mL^−1^ FGF‐basic (Thermo‐Fisher Scientific, 13256‐029). Primary tumor‐infiltrating CD8+ T‐cells were cultured for 1–2 weeks into V‐bottom 96‐well plates (Greiner, 651261) in RPMI‐1640 (Cytiva, SH30027.01) supplemented with 10% human serum (Institut Jacques Boy, Reims, France, 201021334), 0.1% penicillin/streptomycin solution, 1% sodium pyruvate (Thermo‐Fisher Scientific, 11‐360‐070), 30 ng mL^−1^ of rIL‐2 (Gibco, #PHC0021). Primary lung CAFs were cultured into standard culture dishes DMEM/high glucose (Cytiva, SH30243.01) supplemented with 10% FBS (Biosera, FB‐1001/500) and 1% penicillin/streptomycin solution. Additional primary human CAFs from lung adenocarcinoma were purchased from Neuromics (CAF07A) and cultured in MSC‐GRO VitroPlus III, low serum complete medium (Vitro Biopharma, PC00B1).

A549 human lung adenocarcinoma cells (ATCC, CCL‐185) were cultured in DMEM/high glucose supplemented with 10% FBS and 1% penicillin/streptomycin solution. IGR‐Heu large cell carcinoma cells^[^ ^57^ ^]^ were obtained from Fathia Mami‐Chouaib laboratory and cultured in DMEM‐F12 supplemented with 10% FBS, 1% penicillin/streptomycin, 1% of sodium pyruvate, and 1% of Ultroser G (Pall). Jurkat T‐cells were grown in RPMI‐1640 Medium (Gibco, 21875‐034) supplemented with 10% FBS and 1% penicillin/streptomycin. Commercial cell lines were authenticated by short tandem repeat profiling. All cell types were routinely tested for mycoplasma contamination by PCR methods (Eurofins).

### Microfluidic Device Design and Fabrication

The microfluidic device design comprised a central chamber (3.75 mm length × 5 mm width × 0.6 mm height) containing a collagen I gel and two lateral medium compartments (5 mm cube with a 2.5 mm radius half‐cylinder on one side). A needle guide crosses completely the device to make a central channel of 200 µm in diameter. The central chamber was separated from the two lateral ones by sliding walls (20 mm length × 0.48 mm width × 0.63 mm height) which can be retracted once the collagen has polymerized.^[^ ^23^ ^]^ The microfluidic device mold and the sliding walls were fabricated by stereolithography (DWS 028J+, DWS Systems) using the photoresist DS3000 (DWS Systems). Molds and walls were further treated with oxygen plasma (Diener Electronic PICO‐PCCE, 30 s, 0.40 mbar, 100%) and exposed to vapor phase silanization using trichloro(1H,1H,2H,2H‐perfluorooctyl)silane (Sigma‐Aldrich, 448931) for easier removal of the microfluidic chip from the mold and avoid liquid leakage from the lateral chamber, respectively. Mold and walls were extensively washed with ethanol 70% prior to use to prevent cytotoxic effects of residual silane. This silanization step is not mandatory for the walls. Chips were fabricated by casting PDMS (Sylgard 184, Farnell, 101697) at a 10:1 w/w ratio of elastomer to curing agent on the mold equipped with 200 µm Ø microneedles (Serin, No. 3 [Ø 0.20] × 30 mm J‐type). Bubbles were removed with a desiccator before curing the PDMS at 75 °C for 3h to overnight. After peeling from the mold and needle removal, inlets and outlets of a 0.75 mm in diameter were created with a 0.75 mm puncher (World precision instrument, 15453532). Devices were cleaned with ethanol 70% and dried with pressurized air. Any remaining particle was removed from the PDMS surface using transparent adhesive tape (Scotch Tape, 3M). Chips were bound to 0.17 or 1.7 mm thick cover glasses after treatment with oxygen plasma. Sliding walls were immediately placed in position and the chips were placed in a 72 °C oven for 10 min to increase bounding. Surface activation of PDMS for covalent immobilization of collagen was achieved by two alternative techniques: polydopamine^[^ ^81^ ^]^ or (3‐aminopropyl)triethoxysilane (APTES)/glutaraldehyde.^[^ ^82^ ^]^ For the first activation technique, after another round of oxygen plasma treatment, 30 µL of 10 mg mL^−1^ dopamine hydrochloride (Thermo Scientific Chemicals, A11136.06), freshly prepared in 10 mM Tris/HCl buffer pH = 8.5, were added per chip and incubated at room temperature (RT) in the dark for an hour; chips were washed with deionized water (DIW, Millipore). For the second activation technique, a solution of 2% v/v APTES in DIW was added into the central chamber for 30 min at RT; after rinsing with DIW, a solution of 0.1% v/v glutaraldehyde (Sigma‐Aldrich, 340855) in DIW was added and let stand for 15 min at RT; after extensive rinsing with DIW, devices were washed for 48 h to remove any excess of unbound APTES or glutaraldehyde. Devices were then dried by pressurized air, UV sterilized, and stored inside a humidified chamber at 4 °C prior to collagen insertion.

### Microvessel Generation in Collagen Gels

The 200 µm Ø microneedles (Serin, No. 3 [Ø 0.20] × 30 mm J‐type) were coated with 1% w/v bovine serum albumin (BSA, Sigma‐Aldrich, A7906) in PBS for 40 min at RT, before re‐insertion, to prevent collagen from adhering to the needle. Collagen type I gel (high concentration, rat tail, Corning, 354249) was mixed with DIW, 10× PBS (Gibco, 70011–044), and 1 n NaOH (Sigma‐Aldrich, S2770) to a final concentration of 6.5 mg mL^−1^ at pH 7.1–7.4. Collagen solution (14 µL) was then added to fill the central chamber and was let to polymerize in the humid chamber for 10 min at 25 °C and then 20 min at 37 °C. These conditions permitted the formation of thicker collagen fibers, thus promoting a more stable vessel formation.^[^ ^20^ ^]^ After gel polymerization, the lateral chambers were filled with PBS and the sliding walls opened. Then, the needle was gently removed, leaving a tubular opening inside the gel of 200 µm Ø. The resultant tube was filled with 4 µL of 10 µg mL^−1^ human fibronectin (Sigma‐Aldrich, F0895) in PBS and incubated for 30 min at 37 °C. The unbound fibronectin was removed by washes with complete endothelial medium (EGM2‐MV, Lonza, CC‐3156 and CC‐4147). The PBS from the lateral chambers was replaced with medium as well and let to equilibrate overnight under regular cell culture conditions. The remaining holes after needle removal were closed with a drop of semi polymerized PDMS or a silicone glue (Mastic Henkel Loctite Si 5398, Red).

ECs were harvested from the dish and re‐suspended in complete medium containing 4% w/v dextran‐70 kDa (Sigma‐Aldrich, 31390) at 1 × 10^7^ viable cells per mL. 2 µL of cell suspension was gently injected inside the central cell culture channel. Chips were incubated upside down (i.e., inverted at 180°) for 20–30 min inside the incubator to allow cells to attach to the top surface of the channel. Chips were then flipped back and incubated an additional 20–30 min to promote cell adhesion to the bottom surface. Non‐attached cells were removed by gentle washing with medium. Medium was changed 4 h later and a confluent monolayer was allowed to form overnight under static conditions (without flow). Microvessel viability at various time points was evaluated using LIVE/DEAD Cell Imaging Kit (488/570, Invitrogen, R37601).

For the experiments with microfluidic flow, flow rates were controlled by a pressure‐pump (MFCS‐EZ, flow unit M, Fluigent) which was connected to the endothelium chamber inlet via a PTFE tubing (Cole palmer PTFE tubing, 06417‐11), a silicon tubing (Saint‐Gobain, Masterlex 06411–60), and 0.9 mm outer diameter (Metcal, TE720050PK). To achieve a venule/arteriole physiological wall shear stress of 0.1 Pa on the endothelium,^[^ ^83^ ^]^ the flow rate was set at 6.7 µL min^−1^, according to the equation, $\mu =\frac{4\eta Q}{\pi r^{3}}$, where *η* corresponds to the viscosity of the medium at 37 °C, *Q* is the flow rate, and r is the vessel radius.

For the experiments with integration of other TME cell types, cancer cells, CAFs, and CD8+ T‐cells were embedded into the collagen solution at targeted final concentrations of 1000–2700, 500–1000, and 3000 cells per µL, respectively, based on previous on‐chip cell density optimization.^[^ ^63^ ^]^ For the fully autologous vToC experiment, the targeted cell to cell ratios were 3:1 cancer to immune cells and 1:1 cancer to CAF. The medium used for coculture experiment was the EGM2‐MV medium (Lonza, CC‐3156 and CC‐4147) supplemented with 30 ng mL^−1^ rIL‐2.

### Immunofluorescence in Microvessels

For immunofluorescence on 3D microvessels of VE‐cadherin and ZO‐1, solutions were made in DPBS with Ca^++^/Mg^++^ (DPBS++, Gibco, 14040–133). ECs were washed with ice cold DPBS++ and fixed with 4% v/v paraformaldehyde (ThermoFisher, 28906, diluted 1:4) for 20 min at RT and permeabilized with 0.1–0.3% v/v Triton X‐100 (Sigma‐Aldrich, X100) for 30 min at RT. Following a washing step with 4% w/v BSA (Sigma‐Aldrich, A7906), cells were incubated with blocking solution (DPBS ++, 0.1% v/v Tween 20 [Sigma‐Aldrich, P9416], 4% w/v BSA) for 2 h at RT and incubated with anti‐human VE‐cadherin (Rabbit pAb, Abcam ab33168, 1:100 in blocking solution) and anti‐human ZO‐1 (Mouse mAb, Invitrogen ZO1‐1A12, 1:100 in blocking solution) for 1 h at RT. After three 10‐min washes with blocking solution, Alexa 594‐conjugated goat anti‐rabbit IgG (Invitrogen, A11012, 1:1000 in blocking solution) and Alexa 488‐conjugated goat anti‐mouse IgG (Invitrogen, A11001, 1:1000 in blocking solution) were added for 1 h at RT. Nuclei were counterstained with 10 µм Hoechst 33342 (Invitrogen, H1399) for 30 min at RT. Confocal imaging of microvessels was done using a laser scanning microscope (LSM780, Carl Zeiss) equipped with a 40× water immersion objective (Plan‐Apochromat 40×/1.0 NA W) or a 10× objective (EC Plan‐Neofluar 10×/0.3 NA). Approximately 250 consecutive serial sections (each 2 µm thick) were recorded for each microvessel, from which 3D projection images were generated using IMARIS (v9.0.2, Bitplane, USA) or FIJI software.^[^ ^84^ ^]^

For immunofluorescence on 3D microvessels of VCAM‐1, protocol was slightly modified as follows. Solutions were made in DPBS without Ca^++^/Mg^++^ (Euromedex, EU1‐9400‐100). Blocking was in 4% w/v BSA in DPBS. Incubation with anti‐human VCAM‐1 (Mouse mAb 1.4C3, Invitrogen, MA5‐11447, 1:25) was overnight at 4 °C in blocking buffer. After three 10‐min washes with DPBS, 0.1% v/v Tween 20, 4% w/v BSA, the chips were incubated with Alexa 594 goat anti‐mouse IgG (Invitrogen, A11005) and counterstained with 10 µм Hoechst 33342 for 1 h at RT. The chips were then washed with PBS. Confocal Biphoton imaging was done with the Inverted Leica TCS SP8 MP using the HC IRAPO L 25×/1.0 W motCORR objective (Leica cat. 11507704) and the Chameleon Vision II (Coherent) 680 to 1080 nm biphoton laser. VCAM‐1 signal quantification was performed using FIJI software.^[^ ^84^ ^]^ For each cell, the VCAM‐1 fluorescence signal (mean pixel gray value) inside an ellipsoid area (average area = 432 ± 101 µm^2^) centered on the nucleus was measured. Three to four images were acquired per chip. 10 cells per image were measured. For illustration images, only in the Hoechst channel, a mean filter (number of pixels = 3) has been used (FIJI software).

### Live Cell Imaging

Chips were positioned on the motorized stage of an inverted wide‐field fluorescence video‐microscope (Eclipse Ti2‐E, Nikon), enclosed in a Okolab incubation chamber to provide a humid atmosphere with 5% CO_2_ at 37 °C, equipped with a Photometrics Prime BSI Express high sensitivity sCMOS camera and CoolLED Pe4000 light source, and piloted by MetaMorph software. 10× and 20× objectives were used. Phase‐contrast videos were acquired on one to four positions per chip. To capture immune cell dynamics, time intervals of one frame every 1 to 5 min for 30 min to 24 h were chosen, and z stack acquisition mode was used (seven planes every 3 µm). z projections were obtained using the sum or the average projection option in FIJI software. Cells were manually tracked using the manual tracking plugin 2.0.1.jar in FIJI software.

### Quantification of Vascular Permeability

Microvessel permeability was assessed with fluorescein isothiocyanate–dextran 70 kDa (FITC‐Dextran 70, Sigma‐Aldrich, 90719). Chips were positioned on the stage of an inverted wide‐field fluorescence video microscope (DMi8, Leica) enclosed in an incubation chamber to provide a humid atmosphere with 5% CO_2_ at 37 °C. Microvessels were perfused with a solution of 10 µg mL^−1^ of FITC‐Dextran 70 kDa in complete endothelial cell medium at wall shear stress of 0.1 Pa (flow rate 6.7 µL min^−1^). Fluorescent images were acquired every 10 s for 60 min with a 5× magnification objective (N‐plan 5×/NA 0.12 PH 0). To measure the diffusion of Dextran across the microvessel into the surrounding collagen gel, the apparent permeability (*Papp*) in cm s^−1^ was calculated as previously described^[^ ^85^, ^86^ ^]^ based on the following equation: $Pd=\frac{1}{\Delta I}\;\left(\frac{dI}{dt}\right)\frac{r}{2}$, were Δ*I* corresponds to the average fluorescence intensity inside endothelial tube, $\left(\frac{dI}{dt}\right)$ corresponds to the initial rate of increase in fluorescent intensity in the gel and *r* is the vessel radius. The value for $\left(\frac{dI}{dt}\right)$ was measured by plotting the mean gel intensity versus the time and finding the slope during the first 5 min, after linear fitting.

### Leukocyte–Endothelium Adhesion Assay

Jurkat T‐cells were live stained with 5 µм CellTrace Yellow (Life Technologies, C34573) for 20 min at 37 °C, resuspended at a cell concentration of 1 × 10^6^ cells per mL in EGM2‐MV medium and perfused through the endothelial vessels at a wall shear stress of 0.1 Pa (flow rate of 6.7 µl min^−1^) for 10 min using a pressure‐pump (MFCS‐EZ, flow unit M, Fluigent). The Jurkat T‐cells that did not adhere to the endothelium were then washed by applying cell medium alone for another 20 min. Two random fields of view were imaged with an inverted wide‐field fluorescence microscope (DMi8, Leica, 5× magnification) to quantify the number of adherent Jurkat T‐cells per area.

### RT‐qPCR

ECs from the microvessels were dissociated with TryPLE Express (Gibco, 12605010) to obtain intact high‐quality RNA,^[^ ^87^ ^]^ collected by pipetting and homogenized in 500 µL TRIzol Reagent (Invitrogen, 15596026). Total RNA was extracted via double phenol/chloroform extraction and isopropanol precipitation, resuspended in DEPC‐water and quantified using NanodropOne (Thermo Fisher Scientific). The yield was about 150 ng of RNA per microvessel. cDNA synthesis was performed using the high capacity cDNA reverse transcription kit with RNase inhibitor (Applied Biosystems, 4374966), as previously described.^[^ ^25^ ^]^ Multiple‐gene arrays were performed using custom‐designed TaqMan Array cards (ThermoFisher, 4342253). The Taqman probes used are indicated in Figure S1 (Supporting Information). The housekeeping gene *B2M* (coding for β‐2‐microglobulin) was used for normalization or RNA content. For single genes RT‐qPCR, the reactions were performed in duplex using Taqman probes labeled with FAM for the target genes and with VIC for the housekeeping gene *B2M* reference. The Taqman probes used for VCAM‐1 and B2M duplex qPCR quantification were Hs01003372_m1 and Hs99999907_m1, respectively. The results were normalized to B2M and mRNA relative expression levels were calculated using the 2^−ΔΔ^ *^C^* ^t^ method,^[^ ^88^ ^]^ were Δ*C*_T_ = *C*_T,Target_ − *C*_T,Reference_ measured in the same reaction well, and ΔΔ*C*_T_ = Δ*C*_T, Experimental sample_ − Δ*C*_T,Control sample_. 2^−ΔΔ^ *^C^* ^t^ values correspond to target gene mRNA relative expression. Results are expressed in mean value or in log_2_ of mRNA relative expression (2^−ΔΔ^ *^C^* ^t^) according to experiment. Mean value corresponds to the mean of independent vessels for a similar condition.

### RNA‐Seq Analysis

ECs from the microvessels (3D) or from gelatin‐coated 96 wells plastic dish (2D) were dissociated with TryPLE Express (Gibco, 12605010)^[^ ^87^ ^]^ and directly snap freeze in liquid nitrogen. Total RNA isolation was processed using RNeasy micro kit (Qiagen, 74004) including the DNAse Step. Quality controls of the total RNA were assessed using Nanodrop (Thermofisher scientific) for concentration and purity controls, as well as 2100 Bioanalyzer system (Agilent) using RNA 6000 Pico Total RNA kit (Agilent, 5067‐1513) allowing to assess RNA integrity (RIN). 37 to 100 ng of total RNA with RIN ≥9 was sent to sequencing. Samples were sequenced using a Novaseq 6000 system (S1—PE100) and raw data processed using the rawqc Nextflow pipeline (https://doi.org/10.5281/zenodo.8340106). Processed reads were then aligned to the hg38 genome using the RNA‐Seq pipeline (https://zenodo.org/records/13744441). The transcripts per million (TPM) count tables were generated with Salmon 1.10.2.^[^ ^89^ ^]^ As positive control, a pseudo‐bulk was created from the data of the in vivo lung endothelial single‐cell RNA‐Seq atlas^[^ ^9^ ^]^ by summing the feature counts of all cells into a single pseudo sample. Data handling was done using python 3.12.7, all count tables were imported with pandas 2.2.3 and merged in a single dataframe. Features with a total TPM counts <0.1 for all samples were discarded, resulting in a dataframe described by 24 383 features. Signatures were scored using the ssGSEA algorithm.^[^ ^90^ ^]^ The following signatures were used: 13 endothelial signatures^[^ ^9^ ^]^ and eight signatures of fibroblasts and cancer‐associated fibroblasts.^[^ ^61^, ^62^ ^]^ All signatures were gathered in a GMT file and were scored using the python implementation of ssGSEA.^[^ ^91^ ^]^ The resulting normalized expression score matrix was then plotted in a heatmap using seaborn 0.13.2.

For hierarchical clustering, genes with less than ten total count were discarded and the resulting table was normalized using the python implementation of Deseq2.^[^ ^92^ ^]^ The 2000 most variable genes of these sample were selected using the highly variable gene selection method implemented in Scanpy described by Stuart^[^ ^93^ ^]^ on the raw count table, prior to normalization with Deseq2. Clustering of the samples was performed using the AgglomerativeClustering function of sklearn 1.5.2.

### Statistical Analysis

Statistical analyses were performed using the GraphPad Prism (v.9.4) software (San Diego California, USA). Normality of the data were analyzed by Shapiro–Wilk test and statistical significance was determined using parametric (T‐test or One way ANOVA Tukey's test) or non‐parametric (Mann Whitney or Kruskal Wallis) test when appropriate. Asterisks are used to indicate significant differences compared to control *** *p* < 0.001, *** *p* < 0.01, * *p* < 0.05, ns: *p* > 0.05.

## Conflict of Interest

F.M.G. received research support from Roche, Institut Roche and Astrazeneca. All other authors declare no conflict of interest.

## Author Contributions

C.L. and S.L. contributed equally to this work. Conceptualization: C.L., S.L., F.M.G., G.Z., F.S., S.D., M.C.P. Formal analysis: C.L., S.L., N.Z. Funding acquisition: F.M.C., F.M.G., G.Z., F.S., S.D., M.C.P. Investigation: C.L., S.L., G.G., M.N., I.V., S.B., F.S. Methodology: G.G., M.L.N. Resources: S.B., N.P., P.M., A.R., F.M.C., G.Z. Supervision: F.M.G., G.Z., F.S., S.D., M.C.P. Visualization: C.L., S.L. Writing—original draft: C.L., S.L., M.C.P. Writing—review and editing: S.L., P.M., G.Z., F.S., S.D., M.C.P.

## Supporting information



Supporting Information

Supplemental Movie 1

Supplemental Movie 2

Supplemental Movie 3

Supplemental Movie 4

## Data Availability

The data that support the findings of this study are available from the corresponding author upon reasonable request.;
