# Supplementary material for: Bioengineering a Patient‐Derived Vascularized Lung Tumor‐on‐Chip Model to Decipher Immunomodulation by the Endothelium
Source: Adv Healthc Mater. 2025 May 9;14(25):2403446. doi: 10.1002/adhm.202403446 (PMC12477576; doi:10.1002/adhm.202403446)
Supplement: Supplementary file 1 — Supporting Information [file ADHM-14-0-s002.docx]

**SUPPLEMENTARY INFORMATION**

**SUPPLEMENTARY MATERIALS AND METHODS**

**Flow cytometry**

A total of 2.5-5x10^5^ cells in PBS were incubated with 1:500 Zombie NIR (Biologend, 423105) for 20 min at RT in the dark. Cells were washed with FACS buffer (PBS, 2 mM EDTA and 1 % (v/v) human serum (Institut de Biotechnologies Jacques Boy, 020190S)) and incubated with anti-human CD31-PE (Mouse mAb IgG1 k, Biolegend 303106, 1:50) for 25 min at 4 °C in the dark. Cells were washed twice and re-suspended in 150 µl FACS buffer. Flow cytometry was performed using the ZE5 Cell Analyzer (BioRad) and analysis was carried out using FlowJo v10 (Tree Star Inc.). PE Mouse IgG1-k (MOPC-21, BD Biosciences) was used as isotype control.

**Immunofluorescence in 2D**

For immunofluorescence on 2D endothelial monolayers, cells were first seeded in round cover slips pre-coated with 1.5 % (w/v) bovine gelatin (Sigma-Aldrich, G1393). When confluent, cells were washed with ice cold DPBS++, fixed with 4% (v/v) paraformaldehyde for 15 min, permeabilized for 15 min and incubated with blocking solution for 45 min. Cells were stained with anti-human VE-cadherin (Rabbit pAb, Abcam ab33168, 1:100 in blocking solution) and anti-human vWF (sheep pAb, Abcam ab11713, 1:200 in blocking solution) for 1h. Secondary antibodies were Alexa 594-conjugated goat anti-rabbit IgG (Invitrogen A11012, 1:1000 in blocking solution) and Alexa 488-conjugated donkey anti-sheep IgG (Invitrogen A11015, 1:1000 in blocking solution). Nuclei were counterstained using DAPI in mounting media (Invitrogen, p36935). Imaging of 2D samples was done using a wide-field fluorescence microscope (Axioplan 2, Zeiss) equipped with a 40X objective (EC Plan-NEOFLUAR 40x/1.3 NA Oil).**SUPPLEMENTARY FIGURES**


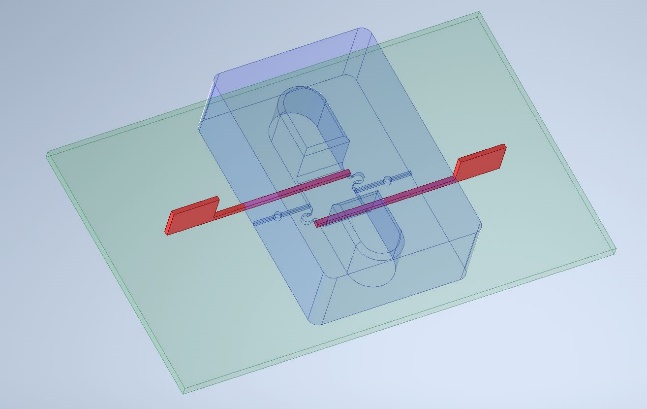

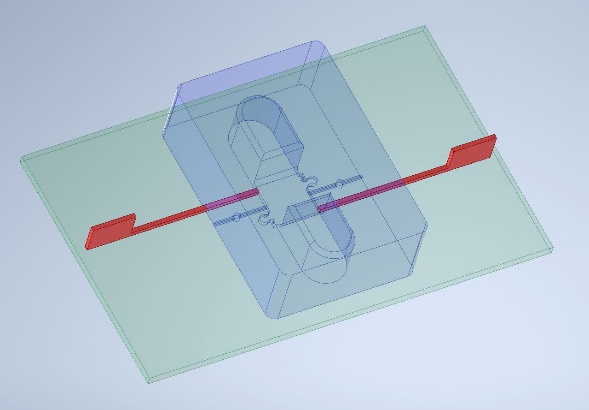


**Supplementary Figure S1: Schematics of the chip design with sliding walls.**

The configuration with closed wall (left) is used for collagen injection and polymerization. After polymerization, walls are slid aside to leave the collagen interface free (right), but not fully removed to avoid leakage.

| **Parameters** | **Patient #2** | **Patient #3** | **Patient #4** | **Patient #5** | **Patient #11** | **Patient #14** |
| --- | --- | --- | --- | --- | --- | --- |
| **Tissue weight** | N/D | 2 g | 5.4 g | 3.66 g | 3.5 g | 4.4 g |
| **(Peritumoral part)** |  |  |  |  |  |  |
| **Number of cells after digestion** | 0.22 x 10^7^ | 2 x 10^7^ | 8.5 x 10^7^ | 6.3 x 10^7^ | 1 x 10^7^ | 3.5 x 10^8^ |
| **Cell viability after digestion** | 80.9 % | 89.30 % | 89.60 % | 71.70 % | 78.00% | 90.00% |
| **Number of cells after CD31+ isolation** | N/D | 0.31 x 10^7^ | 1.25 x 10^7^ | 0.7 x 10^7^ | 4.4 x 10^5^ | 5.6 x 10^6^ |
| **CD31 + cell viability after isolation** | N/D | 68.2 % | 78.3 % | 48.8 % | 90.00% | 87.50% |
| **CD31+ cell purity after amplification** | 98.7 % | 99.1 % | 99.3 % | 99.5 % | N/D | N/D |

**Supplementary Figure S2: Efficiency of endothelial cell isolation.**

For each patient, it is indicated the lung healthy tissue sample weight, the number and viability of cells after mechanic and enzymatic digestion, the number and viability of cells CD31+ enriched after magnetic activated cell sorting (MACS) and finally purity of the CD31+ cells amplified for at least 3 weeks as assessed by flow cytometry. N/D = not determined.


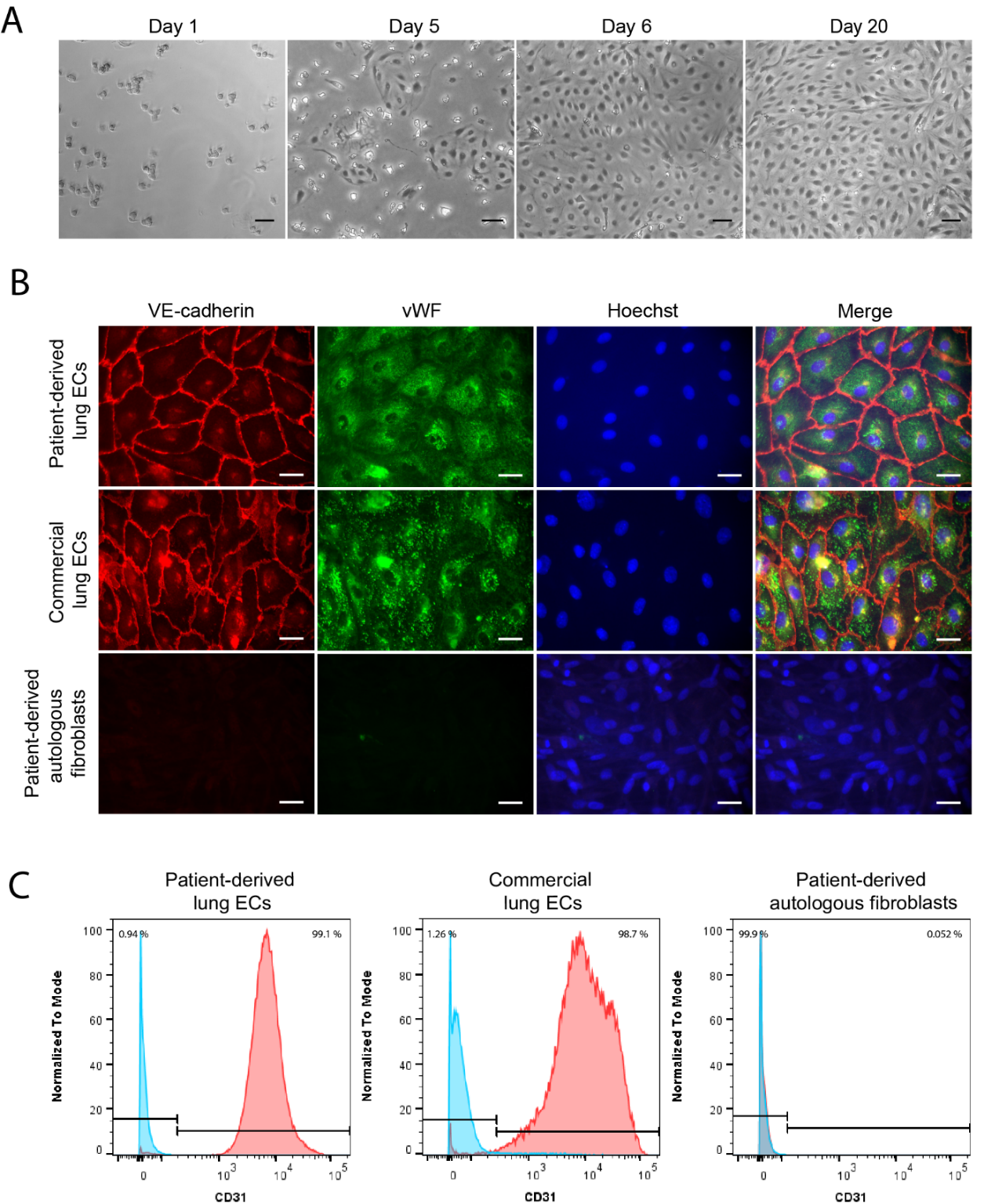


**Supplementary Figure S3**: **Characterization of patient-derived microvascular lung endothelial cells (ECs) by immunofluorescence microscopy and flow cytometry.**

**A.** Representative phase-contrast images of patient-derived lung ECs (patient #4) kept in culture for the indicated days. Scale bars = 100 µm.

**B.** Representative immunofluorescence images of VE-cadherin and vWF staining of primary lung ECs (patient #3). Nuclei were counterstained with DAPI. Commercial lung endothelial cells (HMVEC-L) and freshly isolated autologous fibroblasts served as positive and negative control, respectively. Scale bars = 25 μm.

**C.** Representative CD31 flow cytometry profiles of primary lung ECs (patient #3). The signal obtained with the anti-CD31 antibody is displayed in red, while the isotype control antibody is in blue. The relative percentages of CD31 positive populations are indicated in the diagram. Commercial lung endothelial cells (HMVEC-L) and freshly isolated autologous fibroblasts served as positive and negative control, respectively.


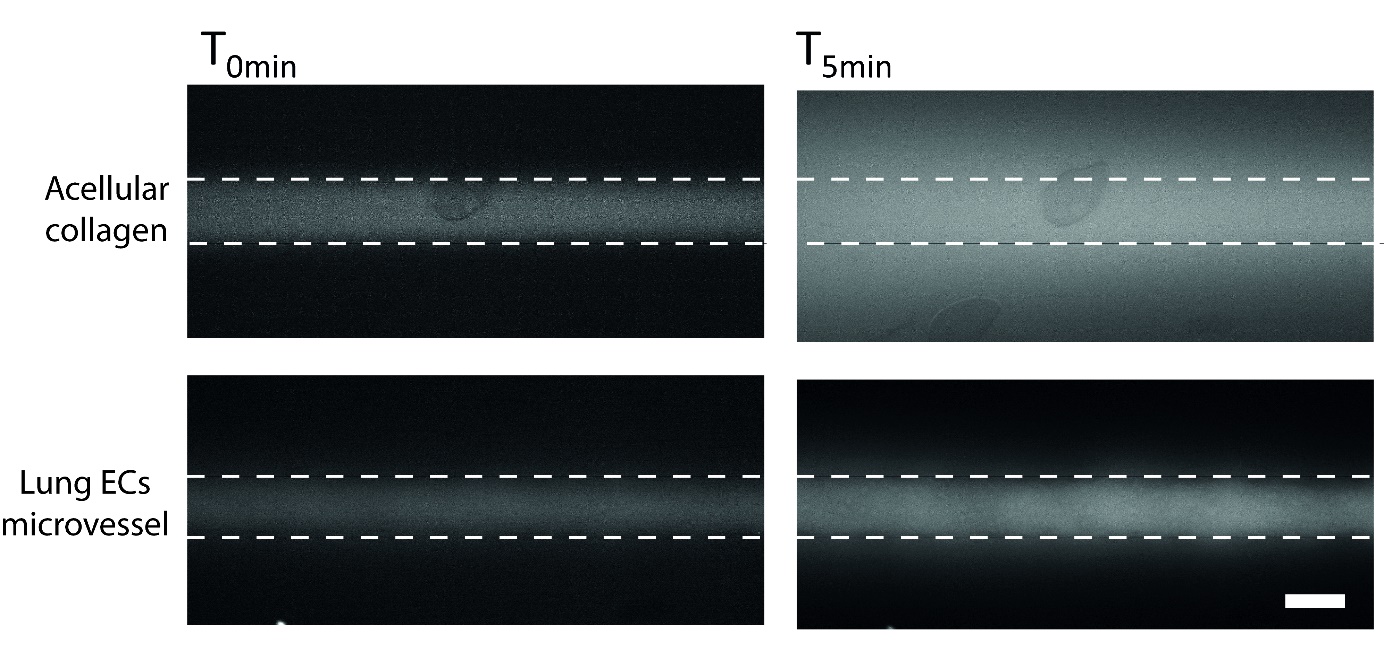


**Supplementary Figure S4: FITC-Dextran permeability test.**

Representative fluorescence microscope images at 0 min and 5 min of permeability tests on acellular collagen or a lung microvessel using 70 kDa FITC Dextran. The dash lines show the limits of the microvessel. Scale bar = 200µm.

**Supplementary Figure S5: Absence of non-endothelial mRNA in RNA extractions from vToC.**

Cell-type specific genes were chosen: PLVAP and vWF for endothelial cells, LUM and DCN for cancer associated fibroblasts, and AZGP1 and KRT19 for cancer cells. The table reports their mRNA mean relative expression (2-ΔΔCt) in the indicated co-culture conditions, with respected to the condition ECs only, as assessed by RT-qPCR. For each condition 1 to 3 independent vessels have been analyzed. No expression detected refers to a cutoff of 40 Ct.

**Supplementary Figure S6: Panel of endothelial genes*.***

Genes were selected for their known contribution to immunomodulation and tumor progression. The corresponding encoded proteins and Taqman probe references used for RT-qPCR are reported.


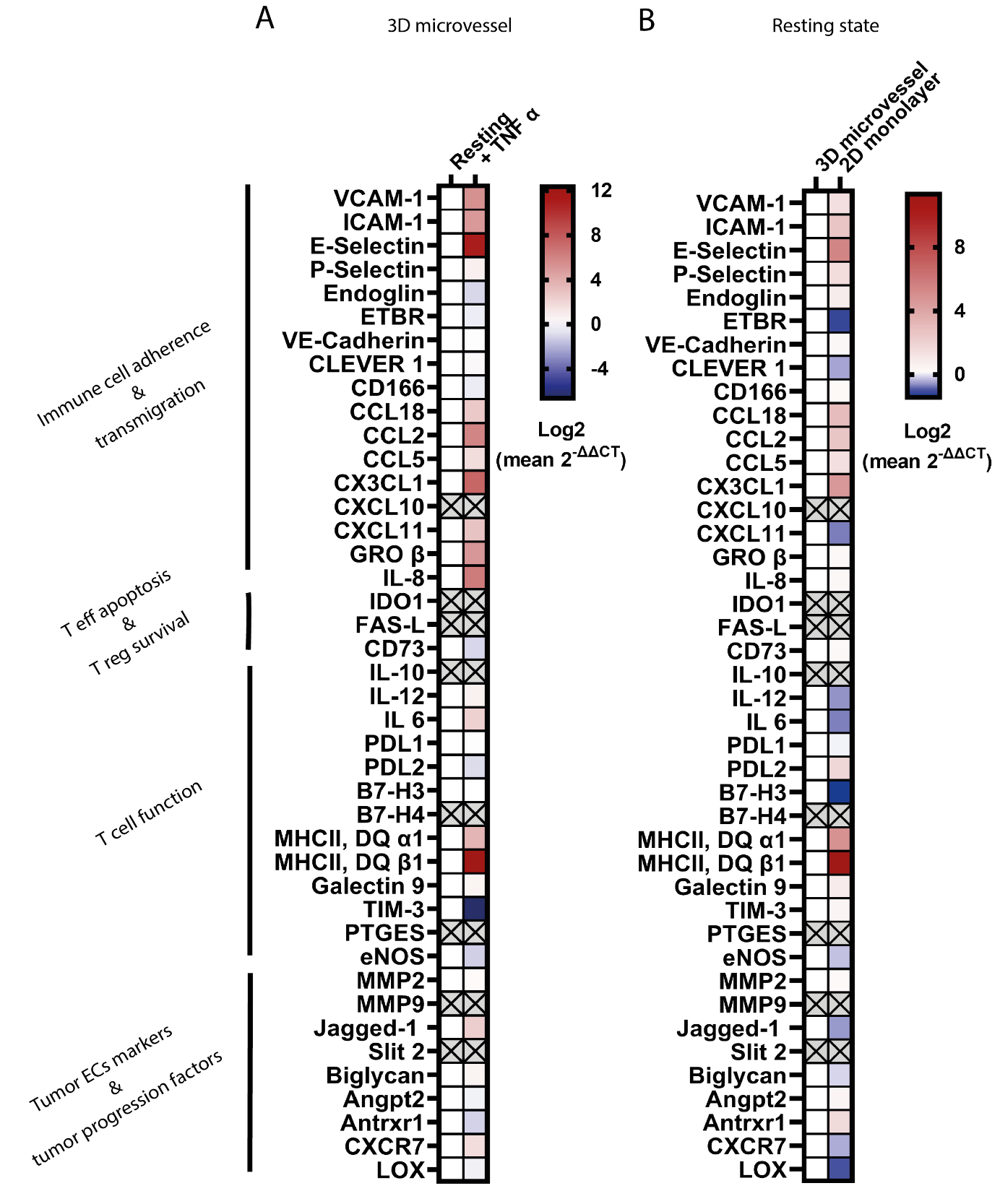


**Supplementary Figure S7: Impact of TNF-α proinflammatory stimulation and endothelium geometry (3D versus 2D) on immunomodulatory functions of the endothelium.**

**A.** Analysis of 42-gene panel expression in patient-derived microvessels (patient #3) upon TNF-α activation. Heatmap reports RT-qPCR analysis of microvessels treated (+ TNF-α) or not (resting) with TNF-α. mRNA relative expression (2^-ΔΔCt^) is reported relatively to control condition (resting) in log_2_ scale.

**B.** Analysis of 42-gene panel expression in 3D patient-derived microvessels (patient #3), as compared to 2D tissue culture. Heatmap reports RT-qPCR analysis of microvessels compared to the same ECs cultured in 2D dishes. mRNA relative expression is reported relatively to control condition (3D) in log_2_ scale.

Gray crossed boxes represent condition where the gene expression was undetected in RT-qPCR using TLDA (Taqman Low density array) technique.


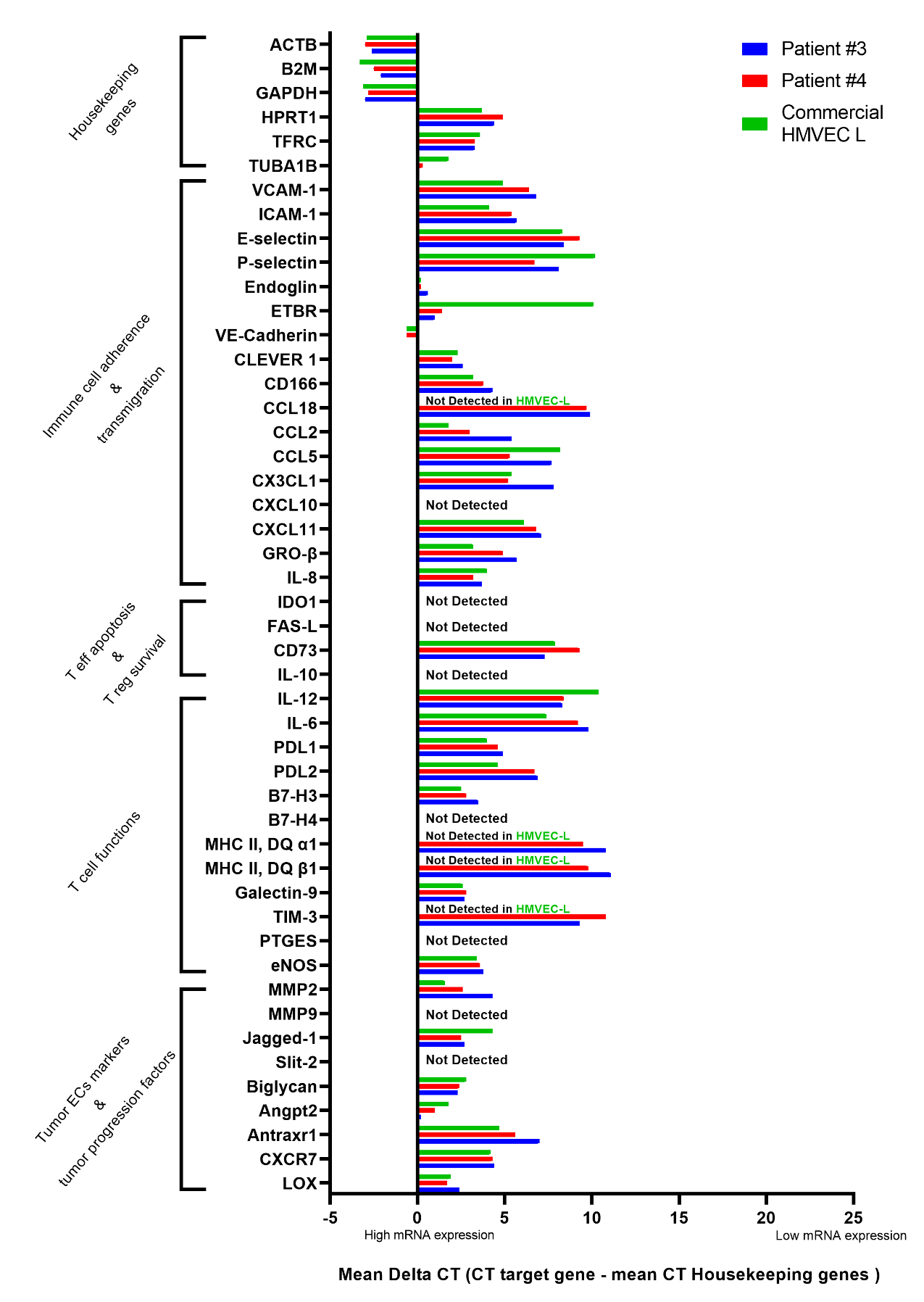


**Supplementary Figure S8: Comparison of ΔCt of the 48 genes in the three endothelial cell types.**

The histograms represent the ΔCt of each gene in the panel, averaged from all samples per same EC type. ΔCt = Ct target gene - mean CT of the 6 housekeeping genes (*B2M, ACTB, GAPDH, HPRT1, TFRC, TUBA1B*). Note the consistency of values between the different EC sources.

**SUPPLEMENTARY MOVIES**

**Movie 1. Confocal imaging 3D projection of a representative microvessel.**

VE-cadherin (red), Hoechst 33342 (blue).

**Movie 2. Autologous vToC, microvessel**

Time length = 90 min. Scale bars = 50 µm.

**Movie 3. Autologous vToC, collagen-embedded cells**

Time length = 495 min. Scale bars = 50 µm.

**Movie 4. Autologous vToC, zoom on cellular dynamics**

Time length = 495 min. Scale bars = 20 µm.
